# Accuracy of computer-guided implantation in the placement of one-piece ceramic dental implants in the anterior region: A prospective clinical study

**DOI:** 10.1371/journal.pone.0237229

**Published:** 2020-09-14

**Authors:** Nopparat Suksod, Chatchai Kunavisarut, Jira Kitisubkanchana

**Affiliations:** 1 Faculty of Dentistry, Mahidol University, Bangkok, Thailand; 2 Faculty of Dentistry, University of North Carolina at Chapel Hill, Chapel Hill, North Carolina, United States of America; 3 Department of Advanced General Dentistry, Faculty of Dentistry, Mahidol University, Bangkok, Thailand; 4 Osaka University Graduate School of Dentistry, Osaka, Japan; 5 Department of Oral and Maxillofacial Radiology, Faculty of Dentistry, Mahidol University, Bangkok, Thailand; National Taiwan University, school of dentistry, TAIWAN

## Abstract

**Purpose:**

Placement of one-piece ceramic dental implants requires precision, which can be enhanced by using a computer-guided system. This prospective clinical study examines the accuracy of partially guided implantation in the placement of one-piece ceramic implants in the anterior region.

**Materials and methods:**

One-piece ceramic dental implants were placed in 20 patients who were missing a central or lateral incisor. Partially guided dental implant placements were performed in all cases. The deviations in the implant positions were analyzed by superimposing post-operative cone beam computed tomography images over pre-operative treatment planning images. The results were reported as deviations (mean ± standard deviation) for three aspects (3D offset, mesio-distal, labio-lingual, and apico-coronal) and in three dimensions (the angle, coronal, and apical parts).

**Results:**

Implants were successfully placed in 20 patients. The mean angular deviation was 4.23±1.84°, whereas the mean coronal 3D offset was 0.98±0.48 mm, and the mean apical 3D offset was 1.57±0.46 mm.

**Conclusions:**

A prospective clinical study involving 20 patients was conducted to measure the accuracy of computer-guided implantation of one-piece ceramic dental implants. Accuracy was determined by comparing the planned implant position to the actual position. Greater accuracy can be expected at the coronal part than at the apical part. The coronal 3D offset was found to be the most accurate.

## Introduction

Since the late 1990s, cone beam computer tomography (CBCT) has become an important diagnostic tool in implant dentistry [1]. CBCT allows dentists to visualize the three-dimensional anatomical structure, particularly of the inferior alveolar nerve, the bony defects, and the maxillary sinus before placing the dental implant. However, placing dental implants in the correct position is usually difficult in conventional implant placement, even with prior CBCT imaging [2].

Computer-guided implantation was introduced to improve diagnosis, treatment planning, and to aid surgery. The use of this technology reduces surgical time and post-operative surgical complications, including pain and swelling resulting from minimally invasive surgery [3]. In addition, complications from the misalignment of dental implants can be significantly reduced by using computer-guided implantation, due to its greater placement accuracy in comparison with freehand placement [4].

Computer-guided implantation uses a surgical template that transfers the implant positioning data from the dental implant planning software to the surgical site. The sequential drilling is performed through the template according to the virtual plan. The implant can be inserted either freehand or by using the surgical template. Freehand implant insertion is defined as partially guided implantation. Some implant systems additionally allow the implant to be inserted through the template; this is defined as fully guided surgery. The results from studies examining the accuracy of computer-guided implantation are varied and difficult to compare, due to the different clinical situations and different experimental procedures involved. A previous systematic review revealed a mean error of 1.12 mm at the entry point and 1.39 mm at the implant apex, and a mean angular deviation of 3.89° [5]. However, the consensus from the International Team for Implantology (ITI) group states that there should be safety margins of 2 mm in relation to the anatomical structure when planning an implant position [6].

Because of the lack of the ability of prosthetic abutments to compensate for misalignment or mis-angulation during surgery, precision is required during treatment planning and placement of a one-piece ceramic implant. Furthermore, the preparation of the abutment can affect the mechanical properties of the material [7, 8]. Therefore, in order to achieve favorable esthetic results and predictable success rates, placement of one-piece ceramic implants demands an experienced surgical and restorative clinician [9].

Using a computer-guided system to insert one-piece ceramic implants may solve the aforementioned problems by enhancing the precision of implant positioning. The aim of this study is to examine the accuracy of using a partially guided computer template in the placement of one-piece ceramic implants for single-tooth replacement in the anterior region.

## Materials and methods

**Fig 1:**
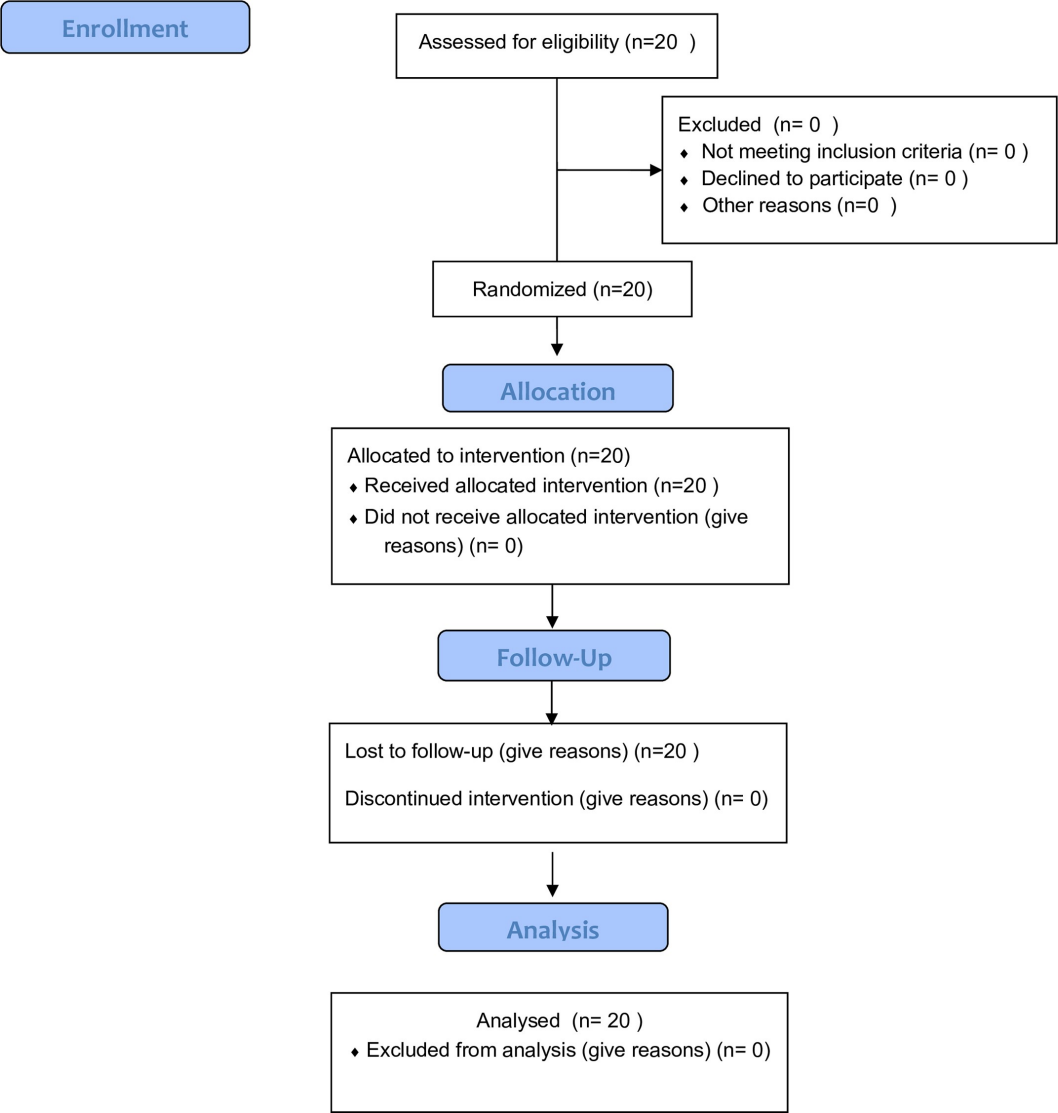
CONSORT flowchart.

This prospective clinical study followed CONSORT guidelines (Fig 1) and was ethically approved by the Institutional Review Board of Mahidol University (COA no. MU-DT/PY-IRB 2016/018.1803). The study was conducted in accordance with international guidelines for human research protection, including the Declaration of Helsinki, the Belmont report, CIOMS guidelines, and the International Conference on Harmonization in Good Clinical Practice (ICH-GCP). Informed consent was obtained from all patients prior to the start of the study. The authors confirm that all ongoing and related trials of this intervention are registered.

### Subjects

The subjects were patients from the Implant Center Clinic, Faculty of Dentistry, Mahidol University, who required dental implant-supported single-tooth prosthesis in the incisor area of the maxilla or mandible. The subjects were enrolled in the study from May 2016–May 2017. They were selected according to the following inclusion and exclusion criteria:

### Inclusion criteria

- Participants had to be between the ages of 20 and 85.
- Participants had to either be healthy or have a well-controlled systemic disease.
- Participants had to have an adequate amount of bone for the stable placement of an implant 3.3 mm in diameter and at least 8 mm in length. However, guided bone regeneration simultaneous with implant placement was allowed.
- Participants had to have a stable occlusal relationship.
- The implant site had to be healthy, without any signs of residual infection.
- It had to be possible to place the one-piece ceramic implant into the proper position according to the prosthesis and the available bone, without abutment preparation.
- The implant site had to have a sufficient bone healing (more than 3 months after tooth extraction).

### Exclusion criteria

- The patient smoked more than 10 cigarettes per day.
- The patient was pregnant.
- The patient had a psychiatric disorder or was unable to give his/her informed consent to participate.
- The patient had a history of radiotherapy of the head and neck region.
- The patient had untreated chronic periodontitis or uncontrolled chronic periodontitis.
- The patient had an inadequate inter-occlusal space for cement-retained restoration.
- The patient had an anterior deep bite occlusion.
- The patient had a history or signs of parafunctional habits, including clenching or bruxism.
- The patient had inadequate bony architecture to obtain primary stability and required major bone augmentation.

Twenty patients requiring implant-supported single-tooth prosthesis in the incisor area of the maxilla or mandible were recruited. Depending on the case, the timing of the dental implant placement protocols was type 3 or 4, according to the types defined by the third ITI consensus conference [10].

**Fig 2:**
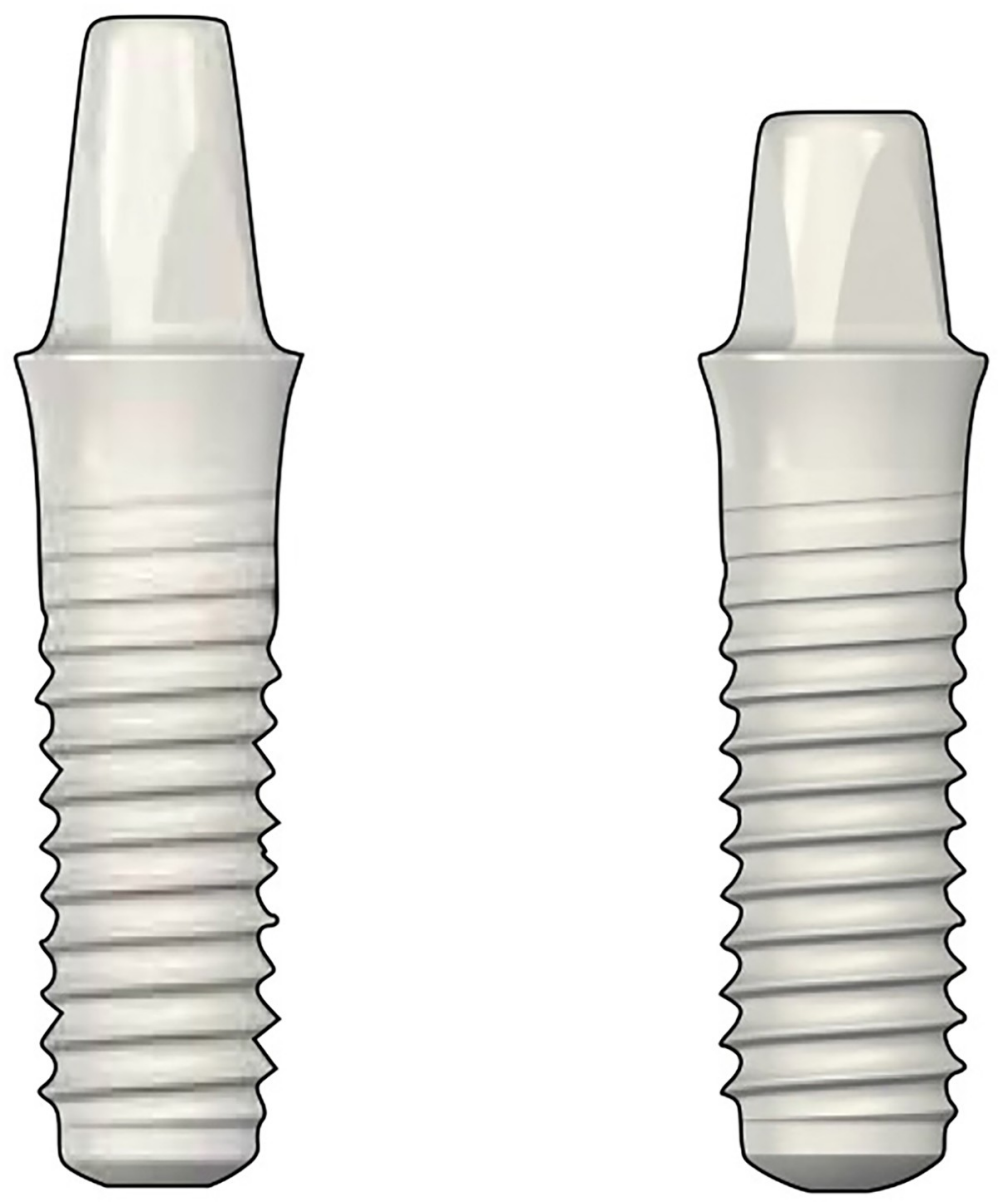
One-piece ceramic dental implants. The one-piece ceramic dental implants had abutment heights of 4 and 5.5 mm.

The implants used in this study were monotype full ZrO_2_ implants (Straumann^®^ PURE Ceramic Implant Monotype, Institut Straumann AG, Basel, Switzerland) with micro-rough surface topographies (Zirconia Large-Grit Sandblasted and Acid-Etched (ZLA^®^) surface). The implants were 3.3 mm in diameter and 8, 10, 12, or 14 mm in length. Two abutment heights (4 and 5.5 mm) were used (Fig 2).

CoDiagnostiX® software, version 9.7 (Dental Wings GmbH and Freiburg, Germany) was used to plan the dental implant, design the surgical template, and evaluate the deviation of the actual implant position from the planned position.

### Preoperative procedure

**Fig 3:**
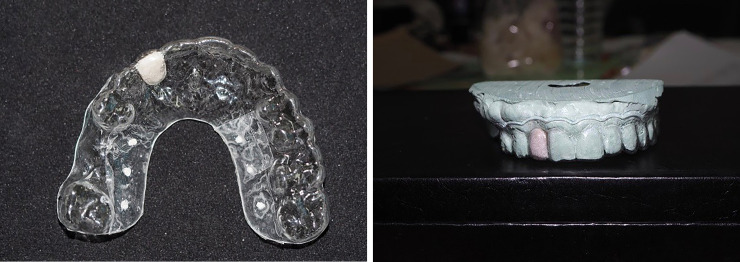
Vacuum radiographic template. (A) Barium sulfate was used as a radiopaque marker around the future prosthesis. (B).

**Fig 4:**
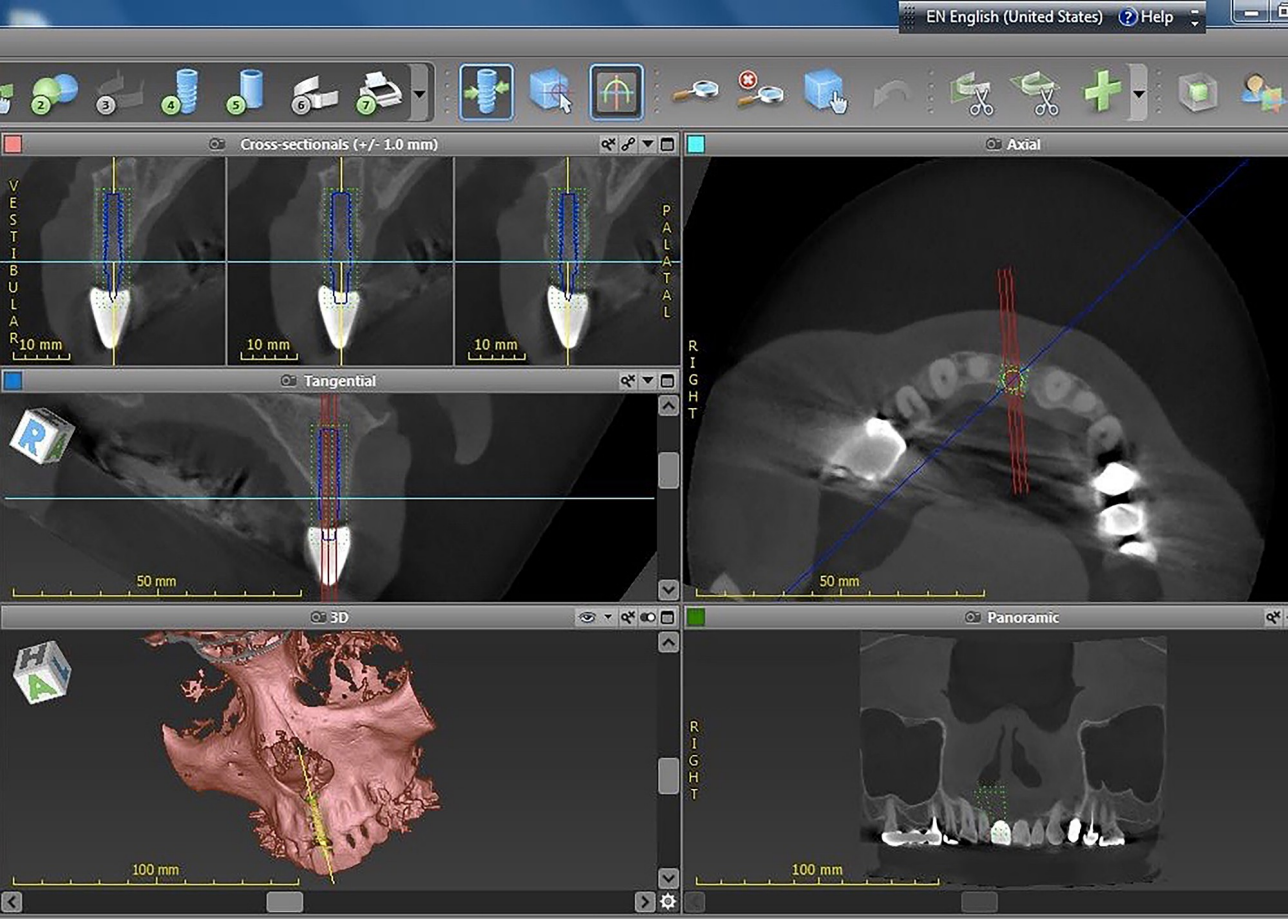
3D virtual image of the Straumann^®^PURE Ceramic Implant. The planned implant positions were created and adjusted using coDiagnostiX^®^ software.

Participants were examined and gave their informed consent. Panoramic radiographs were recorded for each patient. An impression was taken, and then a diagnostic wax-up was created. A vacuum radiographic template with a barium sulfate marker in the area of the future prosthesis was created by the laboratory (Fig 3A and 3B). The diagnostic cast was scanned using a 3D scanner (SHER Aeco-scan 3™) and the output was obtained in Surface Tessellation Language (STL) files. Then, CBCT of each participant was performed using a 3D Accuitomo 170 machine (J. Morita Mfg. Corp., Kyoto, Japan) with an exposure setting of 90 kV, 5 mA, 17.5 s, and a field of view of 10×10 cm, with a voxel size of 0.25 mm. The data were exported as Digital Imaging and Communications in Medicine (DICOM) files. The DICOM and STL files were imported into the coDiagnostiX® software, and then the data were superimposed. Next, the 3D virtual image of the Straumann^®^PURE Ceramic Implant Monotype with a 3.3 mm diameter was inserted, and the placement position was determined. The planned position of the implant was evaluated in every axis by a team of investigators, and adjustments were made to ensure optimal placement of the implants from both the surgical and ideal prosthetic standpoints (Fig 4). Because all implants were 3.3 mm in diameter, 2.8 mm-diameter metal sleeves were selected in all cases.

**Fig 5:**
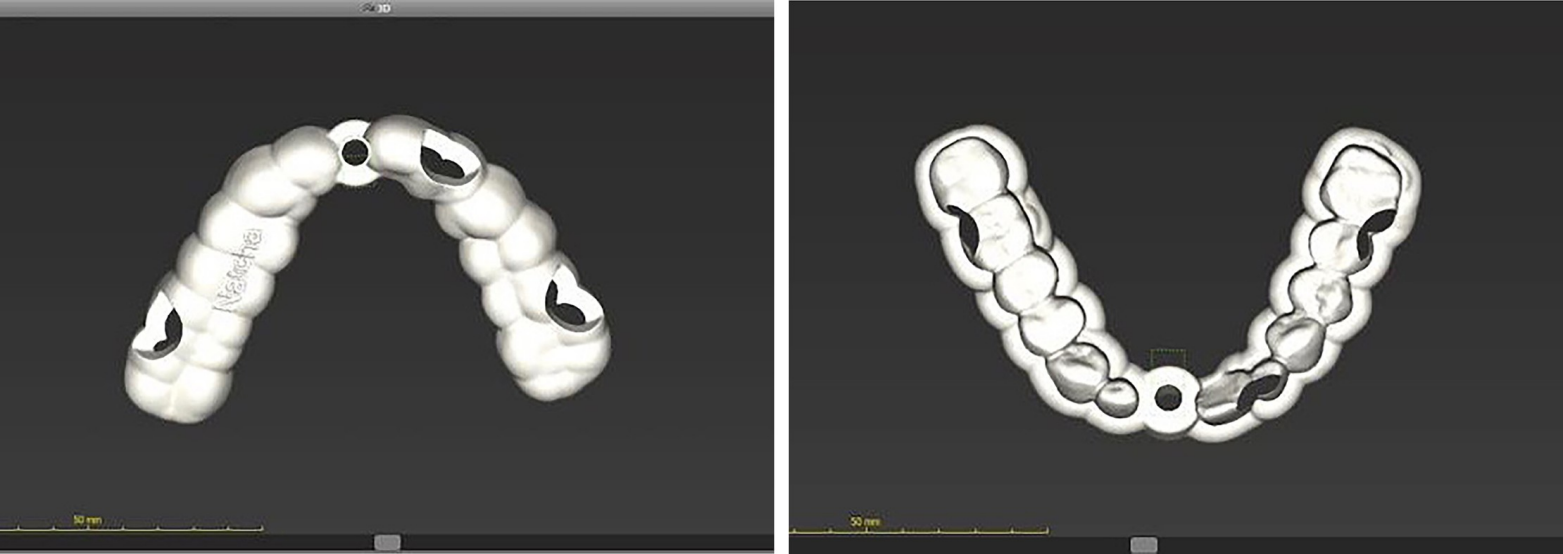
Full-arch coverage design of the digital drill guides. (A) (B).

Digital drill guides were designed (Fig 5A and 5B) and then the virtual templates were printed using a 3D printing device (ProJet™ DP 3000 3-D Production System, SC, USA).

### Implantation

**Fig 6:**
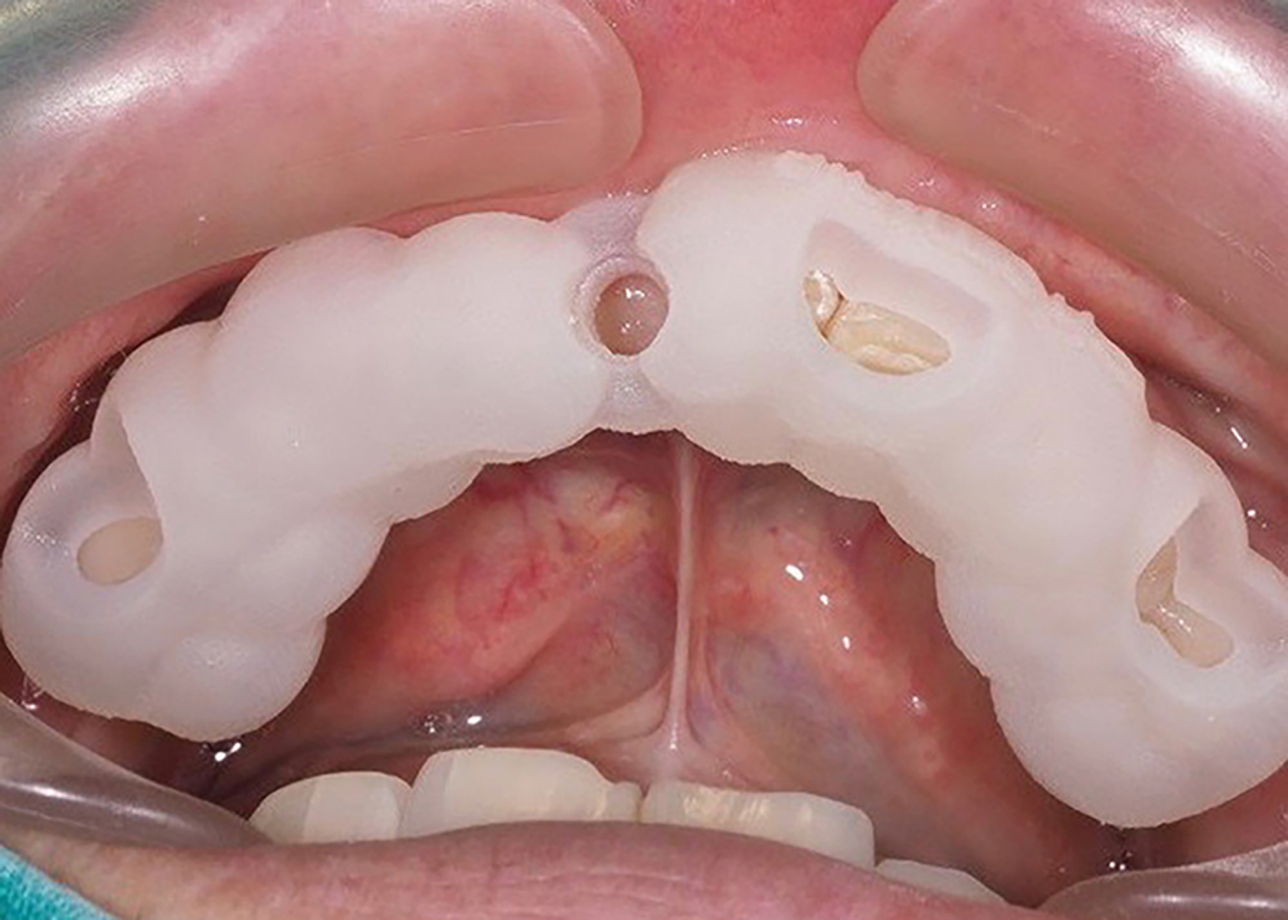
Fit of the computer guided template. The surfaces of the tooth are exposed through the designed window.

**Fig 7:**
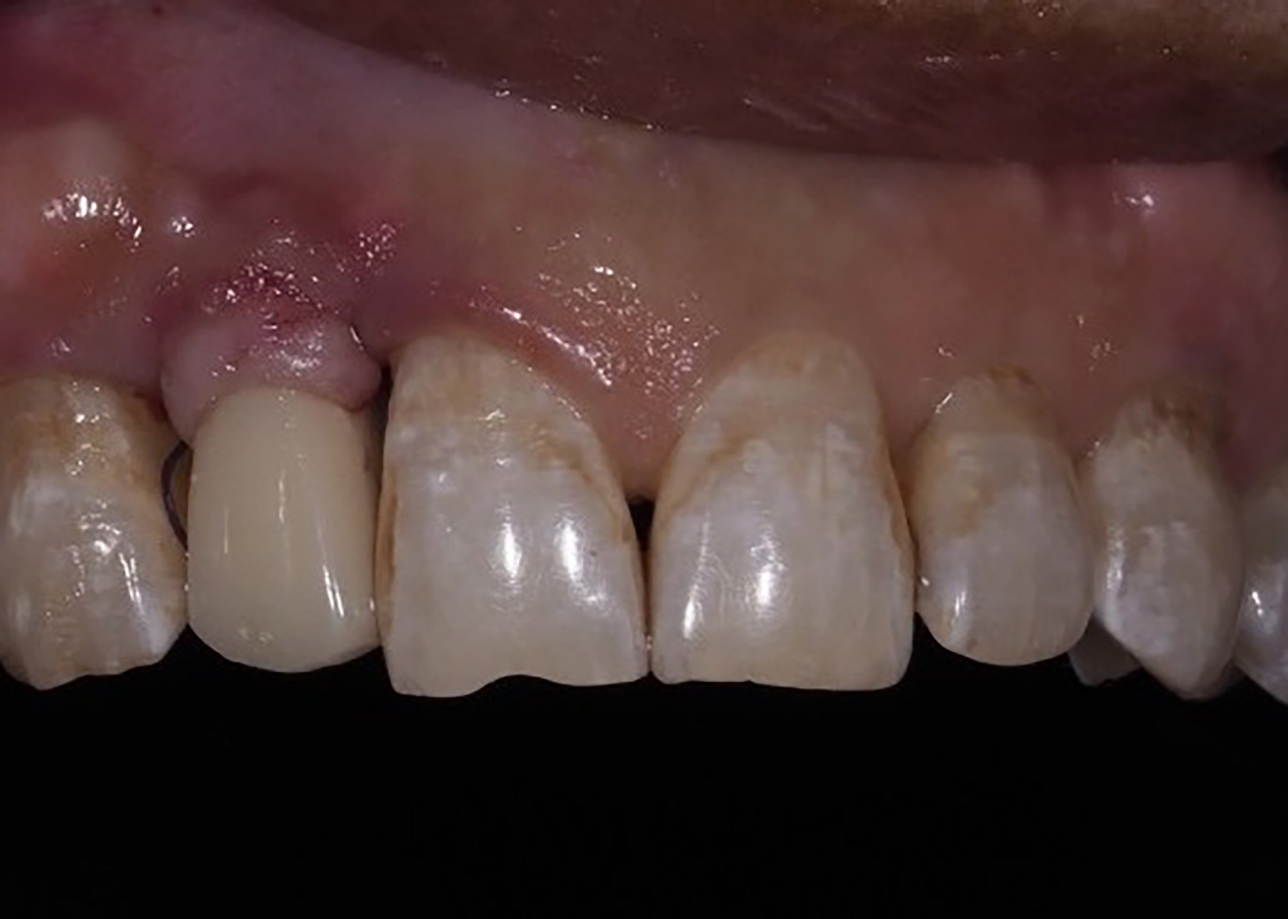
Immediate provisional restoration. The immediate provisional restoration was cemented at the right lateral incisor.

The computer-guided templates were incorporated into the jaws. Windows designed onto the templates allowed the researcher to check the fit (Fig 6). All surgeries were performed under local anesthesia by one surgeon who had adequate experience in computer-guided surgeries. A sulcular through mid-crestal incision was made (a vertical incision was made to prevent excessive tension on the flap), and then a full-thickness flap was created. The implant site was prepared using the Straumann Guided^®^ Surgery kit (Institut Straumann AG, Basel, Switzerland) according to the surgical protocol provided by CoDiagnostiX® software. Because the 2.8 mm-diameter metal sleeve was selected to control the final drill, the one-piece ceramic implant was inserted using a hand piece without the template. If buccal bone resorption was present, then contour augmentation was performed simultaneously using Bio-Oss^®^ (GeistlichPharma, Wolhusen, Switzerland) and Bio-Gide^®^ (GeistlichPharma, Wolhusen, Switzerland). An immediate provisional reconstruction was produced without occlusal loading and was cemented with zinc phosphate cement when a torque of at least 30 N cm was achieved (Fig 7).

### Postoperative procedure

The patients were instructed to rinse twice daily with 0.12% chlorhexidine and to take an antibiotic for 5 days (two tablets of 500 mg amoxicillin, twice daily). An analgesic (400 mg ibuprofen) was also prescribed for two days following the surgery, according to individual need. Patients were advised to follow a soft diet for two weeks following surgery. Two weeks after implantation, the sutures were removed. Periapical and CBCT images were recorded. Postoperative CBCT was performed with the same machine and the same exposure settings as the preoperative CBCT.

### Accuracy measurements

**Fig 8:**
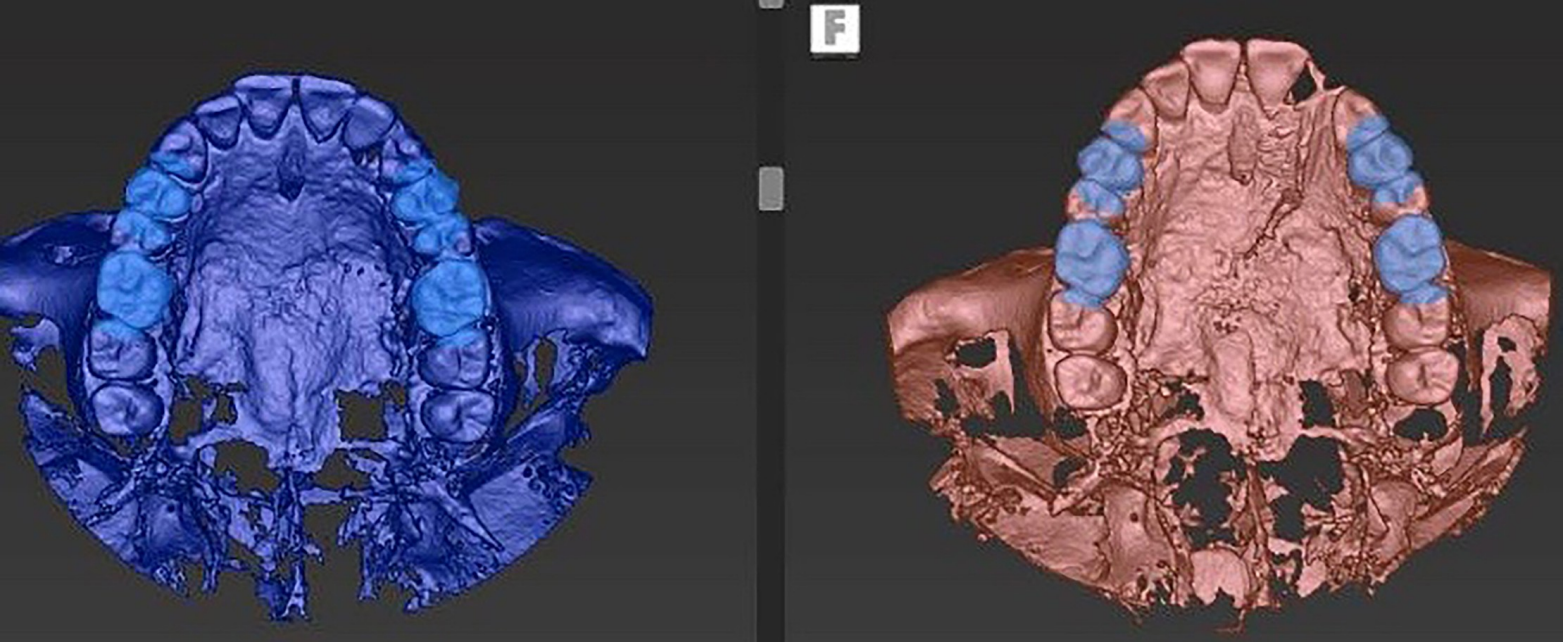
Four anatomical landmarks. The left and right molars and the left and right canines or premolars were used for overlaying the pre-op and post-op CBCT images.

**Fig 9:**
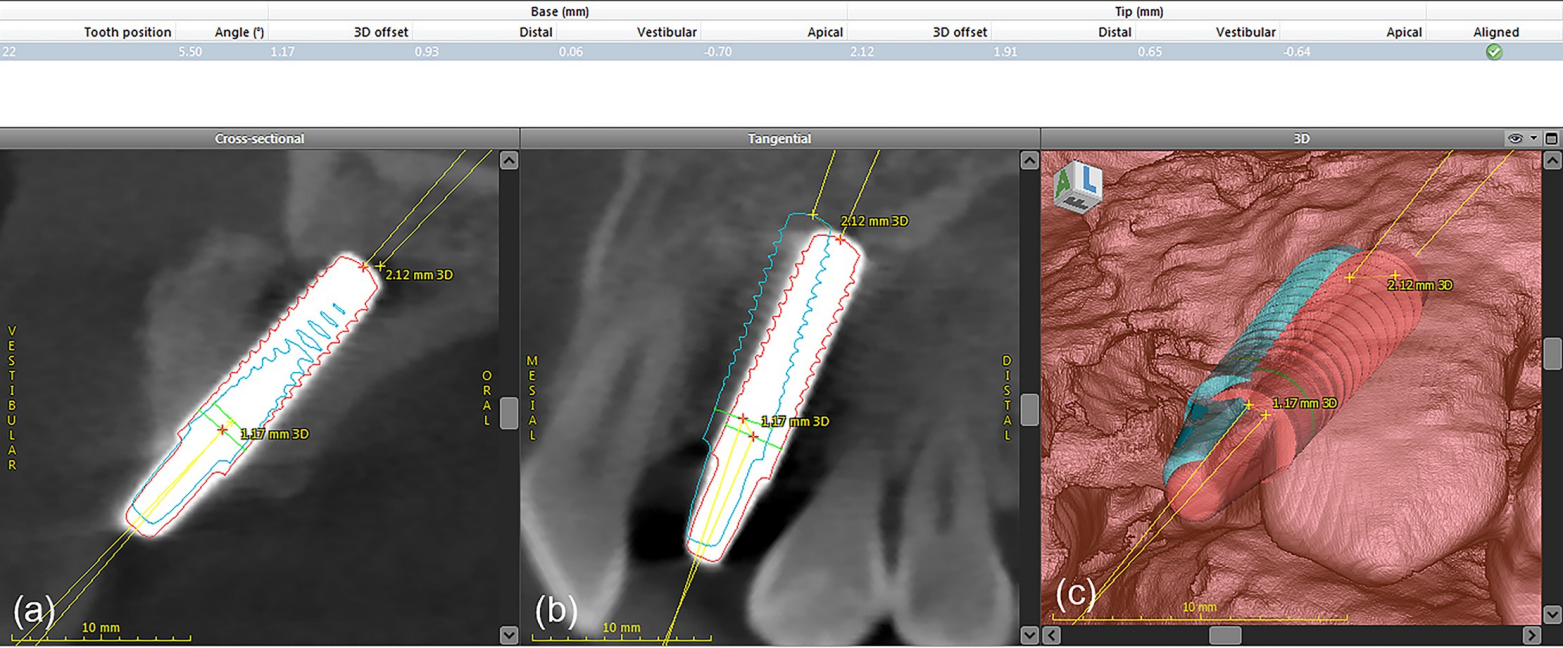
Treatment evaluation tool used for analyzing and reporting the deviations. The coDiagnostiX® treatment evaluation tool calculated and recorded the deviations.

**Fig 10:**
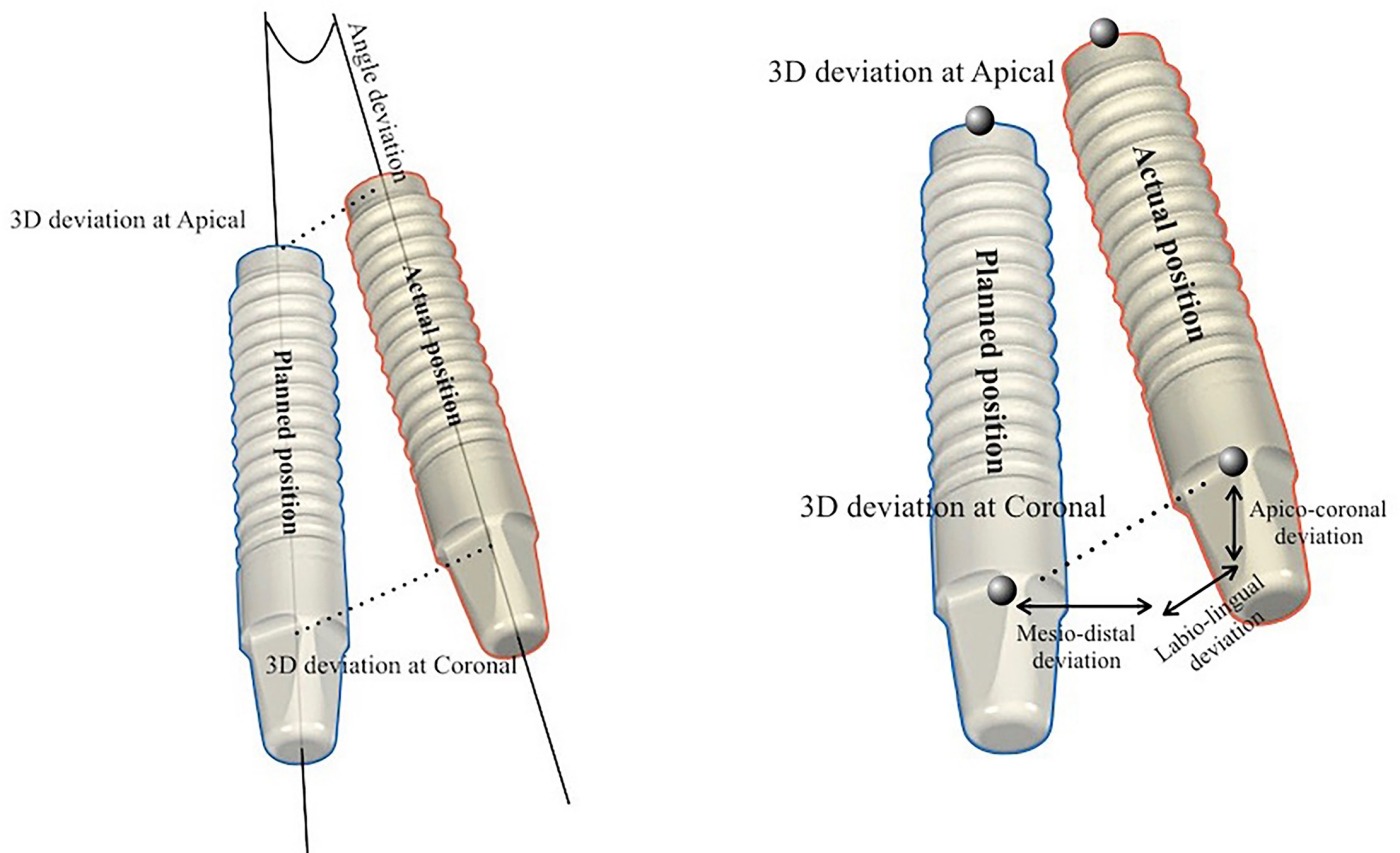
Accuracy measurements. (A) Measurements were recorded in the coronal and apical regions of the implant, similar to those of Kuhl S. et al. (2013) [13]. (B).

The preoperative and postoperative CBCT images were superimposed using treatment evaluation tools included in the coDiagnostiX® software. Four anatomical landmarks (the left and right molars and the left and right canines or premolars) were used for overlaying the CBCT images, with confirmation in the coronal, sagittal, and axial images (Fig 8). Next, the coDiagnostiX® software was used to calculate and record the angular deviation, 3D offset, mesio-distal deviation, labio-lingual deviation, and apico-coronal deviation (Fig 9). The measurements were recorded in the coronal and apical regions of the implant (Fig 10A and 10B), similar to those in the previous study [5]. The CBCT image overlay and all the measurements were performed twice for each patient. The first measurement values were used to represent the study results.

### Prosthetic procedures

Three months after implant placement, a final impression was taken at the abutment level using an impression cap. Polyether (3M™ Impregum™) was used as the impression material, and the crowns were made of either lithium disilicate or zirconia. All-ceramic crowns were cemented to the implant abutment using self-adhesive resin cement (RelyX™ U200; 3M ESPE, St. Paul Minnesota, USA) without any abutment adjustments.

### Statistical analysis

The sample size calculation was performed using the “one sample measurement data” that the previous prospective implemented. Jung et al. 2015 [11] reported that the mean standard deviation of the mean marginal bone loss at the 1-yr follow-up was 0.78 mm and 0.79 mm.

From the formula for sample size analysis:

$$n_{0}=\frac{Z_{\propto }^{2}\sigma^{2}}{d^{2}}$$

The sample size number in this study, at α = 0.05, is at least 15 (let the difference between the population mean and sample mean = 0.4 mm).

The statistical analysis was performed using SPSS^®^ software (SPSS statistics 17.0, Chicago, IL, USA), and the mean and standard deviations were reported for each position. The data for all 20 samples were checked for normality using the Kolmogorov–Smirnov test and were found to have normal distributions. Moreover, each group with dental implant lengths of 10 and 12 mm, respectively, was checked for normality using the Kolmogorov–Smirnov test and was also found to exhibit normal distribution. Therefore, an independent sample t-test was performed to compare the mean deviation between the 10 and 12 mm-long dental implants. P values less than 0.05 were considered to be statistically significant. Pearson correlation was performed for measuring the strength of the correlation between the 3D offset deviation and the mesio-distal, bucco-lingual, and apico-coronal deviations.

## Results

Twenty patients (14 females and 6 males) participated in this study. The mean and standard deviation of the participant age was 49 (12.95) years. No patients experienced any adverse reaction to the one-piece ceramic dental implant. Sixteen dental implants were placed in the maxilla, and four in the mandible. The mean mesio-distal gap in the edentulous area was 7.4 (0.94) mm, and the mean alveolar bone thickness measured at the implant shoulder was 5.33 (1.16) mm. The demographic data for the participants and their edentulous areas are presented in Table 1, while the distribution of each implant according to placement location, length, and abutment height is reported in Table 2. The results of the independent sample t-test showed no significant differences in axis deviation and 3D offset between the 10 mm and 12 mm implants, as presented in Table 3. All the computer-guided templates were stable and achieved an acceptable fit inside the patient’s mouth. Twenty one-piece ceramic dental implants were inserted with an insertion torque of 30–35 N cm. Fourteen implants required simultaneous bone augmentations. Provisional restorations were cemented on eighteen implants, while protective caps had to be placed on the remaining two implants owing to the large amount of bone augmentations. All implant sites healed without any complication. After three months, all implants were restored successfully without any abutment preparations.

**Table 1:** Demographic data for participants and edentulous areas.

	Mean	Std. Deviation	Min	Max
Age (years)	48.5	12.95	24	66
Edentulous space (mm)	7.4	0.94	5	9
Bone thickness (mm)	5.33	1.16	3.5	7.2

**Table 2:** Distribution of one-piece ceramic implants, according to location, length, and abutment height.

Implant Length (mm)			Abutment height (mm)		Total
			4.0	5.5	
10	Tooth position	11	0	3	3
		12	0	3	3
		21	0	1	1
		22	0	1	1
		31	1	0	1
		32	1	1	2
		42	0	1	1
	Total		2	10	12
12	Tooth position	11	0	3	3
		12	0	1	1
		21	0	2	2
		22	0	2	2
	Total			8	8

**Table 3:** Mean and standard deviations in each dimension for 10 mm and 12 mm dental implants.

	Implant length		P value^a^
	10 mm (N = 12)	12 mm (N = 8)	
Angle (°)	4.13 ± 2.18	4.37 ± 1.32	0.78
Coronal (mm)			
3D offset	0.83 ± 0.46	1.22 ± 0.46	0.08
Mesio-distal	0.35 ± 0.27	0.32 ± 0.26	0.77
Labio-lingual	0.36 ± 0.26	0.34 ± 0.16	0.83
Apico-coronal	0.52 ± 0.53	1.04 ± 0.57	0.06
Apical (mm)			
3D offset	1.41 ± 0.48	1.82 ± 0.32	0.06
Mesio-distal	0.76 ± 0.64	0.89 ± 0.55	0.63
Labio-lingual	0.67 ± 0.57	0.92 ± 0.48	0.31
Apico-coronal	0.51 ± 0.52	1.00 ± 0.58	0.06

^a^P-values obtained from independent sample t-tests.

The deviations were reported in three dimensions—the angular deviation, the coronal 3D offset, and the apical 3D offset. The deviations are presented in Table 4. The mean angular deviation was 4.23±1.84°, with maximum and minimum angular deviations of 7.70° and 1.40°, respectively. The mean coronal 3D offset was 0.98±0.49 mm, with maximum and minimum offsets of 1.76 mm and 0.20 mm, respectively. The mean apical 3D offset was 1.58±0.46 mm, with maximum and minimum offsets of 2.27 mm and 0.59 mm, respectively. Similarly, the mean coronal deviations measured in the mesio-distal, bucco-lingual, and apico-coronal aspects were 0.34±0.26 mm, 0.35±0.18 mm, and 0.73±0.59 mm, respectively. The mean apical deviations measured in the mesio-distal, labio-lingual, and apico-coronal aspects were 0.81±0.59 mm, 0.77±0.53 mm, and 0.71±0.59 mm, respectively. Thirteen implants (65% [95% confidence interval (CI): 43.29–81.88%]) were distally deviated, while 14 implants (70% [95% CI: 48.1–85.45%]) were labially deviated, and implants were found to be shallower than planned in 12 cases (60% [95% CI: 38.66–78.12%]). A significantly strong correlation (0.96 correlation coefficient) was observed between the apico-coronal deviation and the 3D offset in the coronal part, as reported in Tables 5 and 6.

**Table 4:** Mean and standard deviations for one-piece ceramic implants in the coronal and apical parts.

	N	Minimum	Maximum	Mean	SD
Axis (°)	20	1.40	7.70	4.23	1.84
Coronal (mm)					
3D offset	20	0.20	1.76	0.98	0.49
Mesio-distal	20	0.03	0.97	0.34	0.26
Labio-lingual	20	0.06	0.74	0.35	0.18
Apical	20	0.03	1.72	0.73	0.59
Apical (mm)					
3D offset	20	0.59	2.27	1.58	0.46
Mesio-distal	20	0.15	2.26	0.81	0.59
Labio-lingual	20	0.11	2.05	0.77	0.53
Apical	20	0.06	1.67	0.70	0.59

SD = standard deviation.

**Table 5:** Correlations between 3D offset and variables in the coronal part.

Variables	Correlations between 3D offset and variables	
	N = 20	
	Pearson Correlation	P-value
Mesio-distal	0.11	0.67
Labio-lingual	0.10	0.68
Apico-coronal	0.96	*<0*.01*

**Table 6:** Correlations in the apical part between 3D offset and variables.

Variables	Correlations between 3D offset and variables	
	N = 20	
	Pearson Correlation	P-value
Mesio-distal	0.39	0.10
Labio-lingual	0.28	0.23
Apico-coronal	0.46	0.05

## Discussion

In this clinical study, 3.3 mm diameter dental implants with 3.5 mm shoulder diameter one-piece ceramic dental implants were successfully placed in 20 patients with anterior edentulous areas. The implants were provisionally restored without any abutment preparation. Based on the deviation measurements, the deviations between the planned and actual positions of the implants were in line with a systematic review by Tahmaseb et al., which reported average deviations of 3.89° in the axial part,1.12 mm in the coronal part, and 1.39 mm in the apical part for 1,854 implants [5]. Additionally, Fürhauser et al. reported mean deviations of 0.84 mm at the implant shoulder and 1.16 mm at the apex, with a mean angular deviation of 2.6° [12]. In this study, 70% of the implants were labially deviated, and 65% were distally deviated; a shallow implant position was found in 60% of the cases. These results were similar to a previous study, in which 70% of the implants were buccally deviated [12]. Based on the results, it can be assumed that partially guided freehand implantation during profile drilling, bone tapping, and implant insertion leads to deviations. Moreover, in all cases, the dental implants were more likely to move toward thinner and softer bony areas; this finding is in agreement with a study by Ozan et al., which reported that bone density and axial deviation in freehand implantation were highly related [13].

Similar findings were observed in relation to the method of implant insertion, where a higher level of accuracy was found when implants were placed through the fully computer-guided template than when they were placed by the freehand technique [5, 14]. This finding is supported by another study that employed the same treatment planning software used in this study to compare the deviations in implant placement between partially guided and fully guided surgery. Fully guided implantations generally showed a higher accuracy, with mean tip and base deviations of 1.54 mm and 1.52 mm, respectively. For partially guided implantations, the values were higher, at 1.84 mm and 1.56 mm, respectively [15].

Another key finding of our study is that similar mean deviations were observed in the coronal part between the labio-lingual and mesio-distal dimensions, which measured 0.34 mm and 0.35 mm, respectively. In contrast, most of the other deviations in this study were related to the depth of the implant (0.73 mm). Significantly strong correlations were observed between the apico-coronal deviation and the 3D offset, particularly in the coronal part. The Pearson coefficient between the apico-coronal deviation and the overall 3D offset was 0.96. In contrast with the mesio-distal and labio-lingual deviation, the coefficients were 0.11 and 0.10; these could indicate a weak relationship to the 3D offset, as presented in Tables 5 and 6. Thus, it can be concluded that the overall 3D offset was most influenced by the apico-coronal deviation. These findings can also be explained by the method of dental implant placement. During the partially guided surgery performed in this study, the templates could only control the implant position in the horizontal plane; the vertical position of the implant depended on the clinician’s perspective. It can be concluded that partially guided implantation was effective in controlling the mesio-distal and labio-lingual deviations of one-piece ceramic implants.

The higher values for deviations measured at the apex were comparable to those in the coronal part in the mesio-distal, bucco-lingual, and apico-coronal aspects, which measured 0.81±0.59 mm, 0.77±0.53 mm, and 0.71±0.59 mm, respectively. This can be explained by the method of implant drilling through the computer-guided template. The greater the distance from the template, the greater the deviation observed, as noted by previous studies reporting higher deviations at the apex in comparison with that at the coronal part [5, 14, 16].

In a computer-guided system, the total deviation is a cumulative error [17, 18]. Metal artifacts from CBCT data can decrease the image quality and distort the outline of anatomical structures [19]. The step of matching the DICOM data with the STL file from the scanned diagnostic cast may also be a possible source of deviation [3]. To avoid such errors in this study, four anatomical landmarks were used by an experienced clinician to revise the superimposed data. However, in a number of cases, difficulties were encountered in distinguishing the anatomical outline; this was because of the patient’s previous metallic restorations and consequent radiographic artifacts that may have affected the matching accuracy. The type of guided support used is another influencing factor. Studies have reported higher accuracy with tooth-supported guides than with mucosa-supported guides, and bone-supported guides show the lowest accuracy [5, 20]. This indicates that the stability of the surgical guides inside the patient’s mouth is important. Vercruyssen et al. reported no difference in the deviation between mucosa-supported and bone-supported guides when the guides were stabilized with fixation screws [21]. Di Giacomo et al. reported that the main factor influencing the deviation was the movement of the surgical template during dental implant preparation [22]. To prevent movement of the guide, only tooth-supported guides with full-arch coverage were used in this study.

In all patients, the final restorations could be delivered without manipulation of the abutment. The predictable esthetic outcome was attributable not only to the precision of the implant placement in three dimensions, but also to the angulation, which was in accordance with the presurgical treatment planning. Regarding the procedure in this study, with freehand insertion of the implant, it can be assumed that the use of computer-guided software for complete treatment planning and surgical template design can lead to errors within a range of “clinical acceptance,” as indicated by the fact that none of the cases required abutment preparation.

Because there was no control group in this study, we could not conclude that the computer-guided surgery was more accurate than the conventional implant surgery when placing one-piece ceramic implants. Although a few studies [23, 24] have reported that the computer-guided surgery provided more accurate implant positioning than the conventional implant surgery, they are based on titanium implants, in which all surgical steps can be completely controlled through surgical templates. To the best of the authors’ knowledge, this study is the first to focus on the accuracy of computer-guided surgery involving a small-diameter one-piece ceramic implant. Studies involving a proper control group and different ceramic implant sizes should be conducted in the future.

## Conclusions

Partially computer-guided implantation can be expected to be more accurate at the coronal part than at the apical part due to the higher deviation in implant position in the apical part than in the coronal part. Moreover, the results indicate that depth deviation is the most deviant dimension, which correlates highly with the overall 3D offset. However, it appears that the errors resulting from the use of computer-guided surgery with partially guided implantation are within a range of “clinical acceptance,” as indicated by the fact that none of the dental implants required abutment preparation and that all were provisionally restored.

Based on the results of this study, the treatment planning distance of 2 mm between the dental implant and vital structures, which is recommended by the consensus from the ITI group [6], may not be sufficient for the placement of one-piece ceramic implants using partially computer-guided surgery. However, further study should be performed to confirm this finding.

## Supporting information

S1 Checklist (DOCX)

S1 File (DOCX)
