# Supplementary material for: Accuracy of computer-guided implantation in the placement of one-piece ceramic dental implants in the anterior region: A prospective clinical study
Source: PLoS One. 2020 Sep 14;15(9):e0237229. doi: 10.1371/journal.pone.0237229 (PMC7489525; doi:10.1371/journal.pone.0237229)
Supplement: S1 File — (DOCX) [file pone.0237229.s002.docx]

**แบบเสนอโครงการวิจัยเพื่อขอรับการพิจารณารับรองจาก**

**คณะกรรมการจริยธรรมการวิจัยในคน ประจำคณะทันตแพทยศาสตร์และคณะเภสัชศาสตร์ มหาวิทยาลัยมหิดล**

**(MU-DT/PY-IRB Submission form)**

1. ชื่อโครงการวิจัย : ผลทางคลินิก ความสวยงาม และความเที่ยงตรงของรากเทียมที่ได้หลังการฝัง one-piece ceramic โดยการใช้ computer guided surgical template
   Title of protocol : Clinical and esthetic outcome of one piece ceramic implants placed with computer guided surgical template

2. ชื่อหัวหน้าโครงการวิจัย: รท.ทพ.ชัชชัย คุณาวิศรุต

Title of investigator: Chatchai Kunavisarut

สถานภาพ ☑ อาจารย์ สังกัด ภาควิชาทันตกรรมทั่วไปขั้นสูง

สถานที่ทำงาน/สถานที่ติดต่อ: ภาควิชาทันตกรรมทั่วไปขั้นสูง คณะทันตแพทยศาสตร์ มหาวิทยาลัยมหิดล

หมายเลขโทรศัพท์ที่ติดต่อได้สะดวก 089-073-7030

e-mail address: drjub@hotmail.com

3. ชื่อนักวิจัยร่วม: 1. ผศ.ดร.ทพญ.จิรา กิติทรัพย์กาญจนา

Assistant Professor Dr. Jira Kitisubkanchana

สถานภาพ ☑ อาจารย์ สังกัด ภาควิชารังสีวิทยาช่องปากและแม็กซิโลเฟเชียล

สถานที่ทำงาน/สถานที่ติดต่อ ภาควิชารังสีสิทยาช่องปากเลกเม็กซิโลเฟเชียส คณะทันตแพทยศาสตร์ มหาวิทยาลัยมหิดล

หมายเลขโทรศัพท์ที่ติดต่อได้สะดวก 081-874-6636

2. ทพญ.นพรัตน์ สุขสด

Miss Nopparat Suksod

สถานภาพ ☑นักศึกษาหลังปริญญา หลักสูตร วิทยาศาสตรมหาบัณฑิต
 สาขาวิชาทันตกรรมรากเทียม(หลักสูตรนานาชาติ)

สถานที่ทำงาน/สถานที่ติดต่อ: ศูนย์ทันตกรรมรากเทียม คณะทันตแพทยศาสตร์ มหาวิทยาลัยมหิดล

หมายเลขโทรศัพท์ที่ติดต่อได้สะดวก 083-543-5669

E-mail address: w.suksod@gmail.com

4. แหล่งทุนสนับสนุนการวิจัย (Funding)

☑ อยู่ระหว่างการขอทุน ☑ ภายนอกมหาวิทยาลัย ระบุผู้ให้ทุน บริษัท Straumann

อยู่ระหว่างการขอทุน โดยบริษัทจะสนับสนุนผลิตภัณฑ์รากเทียม ที่ใช้ในการวิจัยนี้ เมื่อได้รับจดหมายรับรองจากคณะกรรมการจริยธรรมการวิจัยในคน

5. หลักการและเหตุผลที่ต้องทำวิจัย : เนื่องจากในปัจจุบันการทดแทนฟันที่สูญเสียไปด้วยทันตกรรมรากเทียมนั้นเป็นที่แพร่หลายและมีความสำเร็จสูง^[1]^ โลหะไทเทเนียมได้รับการยอมรับว่าเป็น Gold standard ในแง่ของวัสดุที่ใช้สำหรับทำรากเทียม^[2]^ แต่อย่างไรก็ตามในปัจจุบันพบว่าผลทางคลินิกของรากเทียมที่ทำจากโลหะไทเทเนียมเพื่อทดแทนตำแหน่งฟันที่ต้องการความสวยงามนั้น ยังมีข้อจำกัดแง่ของสีของโลหะไทเทเนียมที่สามารถสะท้อนผ่านเหงือกในคนไข้ที่มีเหงือกร่น หรือแม้แต่ในคนไข้ที่มีตำแหน่งของขอบเหงือกปกติ แต่มีลักษณะเหงือกแบบบาง (Thin biotype) ก่อให้เกิดความล้มเหลวของรากเทียมในแง่ของความสวยงาม^[3]^ นอกจากนี้ผลจาก corrosion products ของโลหะไทเทเนียมนั้นยังเป็นที่ถกเถียงกันอยู่ ^[4]^โดยจากการศึกษาพบว่าผู้ที่มีประวัติการแพ้โลหะชนิดใดชนิดหนึ่งนั้นจะมีโอกาสเกิดปฏิกิริยาต่อต้านของร่างกายต่อโลหะไทเทเนียมได้มากกว่าคนปกติ^[5]^ ดังนั้นปัจจุบัน metal-free materials จึงเป็นที่สนใจมากขึ้น ในปัจจุบัน ceramic implant นั้นได้มีการพัฒนาคุณสมบัติทางเคมีและกายภาพให้มีคุณสมบัติที่เหมาะสมสำหรับการทำรากเทียม yttrium-stabilized tetragonal polycrystalline zirconia (Y-TZP) เป็น ceramic ที่ถูกพัฒนาให้คุณสมบัติทนต่อการโค้งงอ ทนต่อการสึกกร่อน และทนต่อการแตกหักได้มากขึ้น^[6-8]^ นอกจากนี้การศึกษาในสัตว์ทดลองยังพบว่าการเกิด osseointegration บนพื้นผิวของ ceramic implant นั้นไม่มีความแตกต่างอย่างมีนัยสำคัญเปรียบเทียบกับ Titanium implant^[9, 10]^ มากไปกว่านั้นมีการศึกษาบ่งชี้ว่าเนื้อเยื่ออ่อนบริเวณรอบ ceramic implant นั้นมีการตอบสนองต่อการหายของเนื้อเยื่อภายหลังการผ่าตัดฝังรากเทียมที่ดีกว่าและพบการอักเสบของเนื้อเยื่อน้อยกว่าเมื่อเทียบกับtitanium implant^[11, 12]^ การศึกษาของ Scarano et al.^[13]^ แสดงให้เห็นว่า ปริมาณจุลชีพที่พบที่พื้นผิวของ ceramic implants มีจำนวนน้อยกว่าอย่างมีนัยสำคัญเมื่อเปรียบเทียบกับพื้นผิวของ titanium implant การศึกษาทางคลินิกของ Payer et al. 2013^[14]^ พบว่าความสำเร็จของ immediate provisional ในการบูรณะฟันหนึ่งซี่โดยใช้ one-piece ceramic implant นั้นไม่มีความแตกต่างอย่างมีนัยสำคัญเมื่อเปรียบเทียบกับการใช้ titanium implant จากการทบทวนวรรณกรรมการใช้โปรแกรมคอมพิวเตอร์เพื่อสร้าง Guided surgical template นั้นจะช่วยลดความคลาดเคลื่อนในการฝังรากเทียมได้ในทุกๆมิติ^[15-18]^ การศึกษาในห้องทดลองพบว่าค่าเฉลี่ยความคลาดเคลื่อนของตำแหน่งรากเทียมเมื่อใช้โปรแกรม coDiagnostiX ร่วมกับการขึ้นรูป surgical template โดยการใช้เครื่องพิมพ์สามมิติ 3D printing เท่ากับ 0.22 mm. (โดยอยู่ในช่วง0.07-0.38 mm.)^[19]^ การฝัง one-piece implant นั้นต้องการความเที่ยงตรงมากกว่าการฝัง two-piece implant เนื่องจากตำแหน่งของรากเทียมนั้นจะมีผลต่อตำแหน่งของครอบฟันโดยตรงไม่สามารถเปลี่ยนแปลงได้ ดังนั้นการผ่าตัดฝัง one-piece ceramic implant โดยการใช้ Computer guided surgical template นั้นจะทำให้มีความแม่นยำของตำแหน่งรากเทียมมากขึ้น

6. วัตถุประสงค์ของการวิจัย : เพื่อศึกษา survival rate, success rate , periodontal status , pink esthetics score, white esthetics score insertion torque และความแม่นยำตำแหน่งรากเทียมเมื่อใช้ computer guided surgical template ในการผ่าตัดรากเทียม ของ one-piece ceramic implant

7. การออกแบบการวิจัย

7.1. ชนิดของโครงการวิจัย

Prospective Clinical Research

7.2. การคัดอาสาสมัคร (Subject selection and allocation) ประกอบด้วย

7.2.1. เกณฑ์การคัดเข้าอาสาสมัคร (Inclusion criteria)

- อาสาสมัครต้องมีอายุ 20-85 ปี
- อาสาสมัครต้องเป็นผู้ที่มีสุขภาพแข็งแรง หรือมีโรคประจำตัวที่สามารถควบคุมได้ดี
- อาสาสมัครเป็นผู้ที่มีความประสงค์จะบูรณะฟันที่หายไปด้วยทันตกรรมรากเทียม และฟันที่หายไปนั้นต้องเป็น implant-supported single restoration ในตำแหน่งฟันหน้า ( Central incisor and lateral incisor )
- อาสาสมัครนั้นจำเป็นต้องมีปริมาณกระดูกเพียงพอที่จะผ่าตัดฝังรากเทียมขนาดเส้นผ่าศูนย์กลาง 3.3 mm. ความยาวของรากเทียมอย่างน้อย 8 mm.ในตำแหน่งที่ต้องการบูรณะด้วยรากเทียม และปริมาณกระดูกนั้นต้องสามารถทำให้รากเทียมมี primary stability
- อาสาสมัครต้องมีรูปแบบการสบฟันที่เสถียร และมีฟันกรามหลังอย่างน้อย 1 คู่ในแต่ละฝั่ง

ตำแหน่งที่จะทำการผ่าตัดฝังรากเทียมต้องไม่มีภาวะติดเชื้อของเนื้อเยื่อหลงเหลืออยู่

7.2.2. เกณฑ์การคัดออกอาสาสมัคร (Exclusion criteria)

- มีประวัติสูบบุหรี่มากกว่า 10 มวนต่อวัน
- กำลังตั้งครรภ์
- ผู้ที่เคยมีประวัติได้รับการฉายแสงบริเวณขากรรไกรและใบหน้า หรือมีประวัติได้รับยาในกลุ่ม bisphosphonate
- ผู้ที่มีสภาวะจิตใจไม่ปกติ ไม่สามารถดูแลสุขภาพช่องปากได้ตัวตัวเอง
- มีสภาวะติดเชี้อเฉียบพลัน( Acute infection ) ในบริเวณที่จะผ่าตัดฝังรากเทียม
- ผู้ที่มีภาวะ Chronic periodontitis ที่ไม่ได้รับการรักษา หรือไม่สามารถควบคุมโรคได้
- มีรูปแบบการสบฟันแบบ deepbite ในตำแหน่งที่จะทำการผ่าตัดฝังรากเทียม
- มีประวัติการนอนกัดฟัน หรือมี para-functional habits

7.2.3. เกณฑ์การยุติการอาสาสมัคร (Subject withdrawal criteria)

- ภายหลังการผ่าตัดฝังรากเทียมพบว่ารากเทียมที่ฝังไม่มี primary stability
- อาสาสมัครขาดการติดต่อและไม่ประสงค์มาตามนัดติดตามผล
- อาสาสมัครมีความประสงค์ที่จะออกจากงานวิจัย

7.2.4. เกณฑ์การยับยั้งหรือยุติโครงการวิจัย (Study termination criteria)

- เมื่อพบว่าภายหลังการฝังรากเทียมเกิดผลไม่พึงประสงค์แก่อาสาสมัคร เมื่อตรวจสอบแล้วพบว่ามีผลมาจาก one-piece ceramic implant ที่ใช้

7.3. การคำนวณขนาดตัวอย่าง (Sample size calculation) :จากการทบทวนวรรณกรรมพบว่า การศึกษาของ Jung et al. 2015^[20]^ ได้รายงานผลค่าเฉลี่ยการละลายตัวของ marginal bone 1 ปีภายหลังการฝัง one-piece ceramics implant เท่ากับ 0.78 โดยมีค่าความเบี่ยงเบนมาตรฐานเท่ากับ 0.79


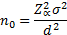


โดย Z_α_ = 1.96 (ใช้ค่าอัลฟาที่ระดับความเชื่อมั่น 0.05)

σ = 0.79 (จากstandard deviation การละลายของ marginal bone ในงานวิจัยของ Jung et al 2015^[1]^
d = 0.4

จำนวนอาสาสมัครที่คำนวณได้อย่างน้อย = 15 implants

7.4. จำนวนอาสาสมัครโครงการวิจัย (Sample size) : 20 implants

☑ Healthy volunteers

7.5. การดำเนินการหากอาสาสมัครถอนตัวออกจากการวิจัย :เมื่อมีอาสาสมัครถอนตัวจากงานวิจัยน้อยกว่า 5 รายจะไม่มีการหาผู้เข้าร่วมการวิจัยเพิ่มเติม เนื่องจากมีการชดเชย drop out sample ไว้แล้วจำนวน 5 ราย

8. กระบวนการวิจัย : อาสาสมัครที่เข้าร่วมโครงการวิจัยจำเป็นต้องมาตามนัดจำนวน 7 ครั้ง แต่ละครั้งใช้เวลาประมาณ 30-40 นาที

| ขั้นตอนการรักษาปกติ | ขั้นตอนการวิจัยที่เพิ่มเติมมาจากขั้นตอนการรักษาปกติ |
| --- | --- |
| 1.ตรวจสภาพช่องปากและถ่ายภาพรังสีปริทัศน์หรือภาพรังสีส่วนตัดอาศัยคอมพิวเตอร์ชนิดโคนบีม | 1.ตรวจสภาพช่องปากเพิ่มเติม ตรวจการสบฟันโดยละเอียดและภาพรังสีส่วนตัดอาศัยคอมพิวเตอร์ชนิดโคนบีมเพื่อนำไปประกอบการวางแผนการรักษาโดยใช้คอมพิวเตอร์ |
| 2. การวางแผนการรักษา | 2. วางแผนตำแหน่งการฝังรากเทียมโดยใช้โปรแกรม coDiagnostiX software และนำข้อมูลที่ได้มาขึ้นรูป surgical template โดยใช้เป็นแนวทางสำหรับการผ่าตัดฝังรากเทียม |
| 3.การผ่าตัดฝังรากเทียม   - รากเทียมที่ใช้จะทำจากโลหะไทเทเนียมที่มีส่วนประกอบสองส่วน ได้แก่ส่วนราก (fixture) และส่วนตัวค้ำครอบฟัน (Abutment) - จะกระทำการผ่าตัดภายใต้ยาชาเฉพาะที่ โดยตำแหน่งที่ทำการฝังจะเป็นไปตามตำแหน่งที่ได้วางแผนไว้โดย การขยายขนาดกระดูกกระทำโดยมือเปล่า และเป็นไปตาม surgical protocol ของแต่ละชนิดและเต่ละบริษัทรากเทียม - เมื่อทำการฝังส่วนรากลงไปในกระดูกและมี insertion torque มากกว่าหรือเท่ากับ 35 Ncm. จะพิจารณาบูรณะด้วยครอบฟันชั่วคราวในกรณีที่คนไข้มีความต้องการ หรือพิจารณาใส่ healing abutment ร่วมกับ provisional prosthesis อื่นๆได้แก่ acrylic removable plate หรือ resin bonded fixed prosthesis เป็นต้น ในกรณีที่เมื่อฝังรากเทียมแล้วมี insertion torque ต่ำกว่า20 Ncm.จะทำการใส่ cover screw และเย็บเหงือกปิด`. - การปรับแต่ง provisional prosthesis นั้น จะออกแบบให้ไม่กัดสบโดนกับฟันคู่สบและประชิดกับฟันซี่ข้างเคียงเพียงเล็กน้อย - หลังจากการผ่าตัดคนไข้จะได้รับการถ่ายภาพรังสีรอบปลายรากภายหลังการฝังทันที | 3.ก่อนเริ่มการผ่าตัดจะนำ computer guided surgical template ที่ขึ้นรูปมาลองในช่องปากเพื่อทดสอบความ stable ของ template   - รากเทียมที่ใช้คือ one-piece Straumann® PURE Ceramic Implant การฝังรากเทียมจะกระทำตาม surgical protocol ของบริษัท ภายขยายขนาดกระดูกกระทำภายใต้ computer guided surgical template ภายหลังการฝังจะวัด insertion torque โดยใช้ Ratchet with torque control device - รากเทียมที่มี insertion torque มากกว่าหรือเท่ากับ 35 Ncm. จะทำการใส่ครอบฟันชั่วคราวและยึดติดด้วย temporary cement. ในรายที่มี insertion torque น้อยกว่า 35 Ncm. จะใส่ protective cap เพื่อป้องกัน abutment และการเจริญของเหงือกโดยรอบ - การปรับแต่งให้ครอบฟันชั่วคราวนั้นจะออกแบบให้ครอบฟันชั่วคราวไม่กัดสบโดนกับฟันคู่สบและประชิดกับฟันซี่ข้างเคียงเพียงเล็กน้อย - หลังจากการผ่าตัดอาสาสมัครจะได้รับการถ่ายภาพรังสีส่วนตัดอาศัยคอมพิวเตอร์ชนิดโคนบีม ทันทีหรือหลังจากการผ่าตัดฝังรากเทียมไม่เกิน 1 สัปดาห์เพื่อนำมาเปรียบเทียบความเบี่ยงเบนของตำแหน่งรากเทียมตำแหน่งจริงกับตำแหน่งที่ได้วางแผนไว้ในโปรแกรม |
| 4. การใส่ครอบฟัน การใส่ครอบฟันจะภายหลังการฝังรากเทียม 3 เดือน โดยครอบฟันที่ใช้จะเป็นครอบฟันชนิด All ceramics crown หรือ porcelain fuse to metal crown จากนั้นทำการถ่ายภาพรังสีรอบปลายรากฟันภายหลังการใส่ครอบฟันทันที   - ภายหลังการผ่าตัด 14 วันผู้ป่วยจะได้รับการนัดหมายเพื่อมาทำการตัดไหม ตรวจประเมินแผลผ่าตัด และปรับแต่งการสบฟันให้สัมผัสเพียงเล็กน้อยกับ ฟันคู่สบ | 4. การใส่ครอบฟันจะทำภายหลังการฝังรากเทียม 3 เดือน โดยครอบฟันที่ใช้จะเป็นครอบฟันชนิด All ceramics crown ที่ขึ้นรูปโดยเทคนิค CAD-CAM และยึดครอบฟันด้วย cements จากนั้นทำการถ่ายรังสีรอบปลายรากภายหลังการใส่ครอบฟันทันที   - ภายหลังการผ่าตัด 14 วันผู้ป่วยจะได้รับการนัดหมายเพื่อมาทำการตัดไหม ตรวจประเมินแผลผ่าตัด และปรับแต่งการสบฟันให้สัมผัสเพียงเล็กน้อยกับ ฟันคู่สบ |
| 5. การติดตามผล   - 6 เดือนโดยทำการตรวจประเมินเหงือกบริเวณที่ฝังรากเทียมโดยการ ตรวจร่องลึกปริทันต์ที่เกิดขึ้น มีการถ่ายภาพรังสีรอบปลายรากเพื่อประเมินระดับกระดูกรอบรากเทียม - 12 เดือนทำการตรวจประเมินเหงือกบริเวณที่ฝังรากเทียมโดยการ ตรวจร่องลึกปริทันต์ที่เกิดขึ้น มีการถ่ายภาพรังสีรอบปลายรากเพื่อประเมินระดับกระดูกรอบรากเทียม | - 5. นัดติดตามผลภายหลังการใช้งาน 6 เดือนโดยตรวจ periodontal status ได้แก่ probing depth , clinical attachment level , recession และมีการถ่ายภาพรังสีรอบปลายรากฟัน เพื่อประเมินระดับกระดูกโดยรอบรากเทียม - 12 เดือนโดยตรวจ periodontal status ได้แก่ probing depth , clinical attachment level , recession และมีการตรวจประเมิน esthetic outcome โดยใช้ pink esthetic score ,white esthetic scoreและมีการถ่ายภาพรังสีรอบปลายรากฟัน เพื่อประเมินระดับกระดูกโดยรอบรากเทียม |

9. สถานที่ทำวิจัย

☑ Single center : คณะทันตแพทยศาสตร์ มหาวิทยาลัยมหิดล

10. การส่ง **Specimen** ออกนอกมหาวิทยาลัยมหิดล ☑ ไม่มี

11. ระยะเวลาที่ทำวิจัย : มีนาคม 2559 – มีนาคม 2561

12. กระบวนการเก็บข้อมูล (Data collection process) : ใช้แบบบันทึกข้อมูล

โดยแบบบันทึกข้อมูลนั้นมีรายละเอียดได้แก่

1. ประวัติทั่วไป โรคประจำตัว ยาที่ได้รับในปัจจุบัน
2. ข้อมูลเกี่ยวกับสภาวะของเนื้อเยื่อปริทันต์และลักษณะการสบฟัน ก่อนการผ่าตัดฝังรากเทียม และภายหลังการใช้งานใน 6 เดือนและ1ปี ตามลำดับ
3. ความยาวและขนาดของรากเทียมที่ใช้
4. Insertion torque ที่ได้ภายหลังการฝังรากเทียม

13. การวัดผล/การวิเคราะห์ผลการวิจัย (Outcome measurement/Data Analysis)

- ผลลัพธ์หลัก (Primary outcome)

ศึกษาถึงการคงอยู่ของ one-piece ceramic implant ในกระดูกภายหลังการใช้งานในเวลา 1 ปี (survival rate)

- ผลลัพธ์รอง (Secondary outcome)

- ศึกษาถึงการละลายของกระดูกรอบ one-piece ceramic implant ใน1ปี โดยการใช้ standardized
periapical radiograph เปรียบเทียบระหว่างภายหลังการฝังรากเทียม หลังจากใช้งาน 6 เดือน และหลังจากใช้งาน 1 ปี

- ศึกษาถึงของ success rate ภายใน 1 ปีของ one-piece ceramic implant ตาม criteria ของ Buser et al. 1990^[2]^ ได้แก่

1. บริเวณที่ฝังรากเทียมไม่มีอาการปวด ชา หรือความรู้สึกผิดปกติของการรับรสภายหลังได้รับการผ่าตัดฝังรากเทียม
2. บริเวณเนื้อเยื่อรอบๆรากเทียมไม่พบว่ามีสัญญาณของการติดเชื้อ และไม่พบการเกิดขึ้นของหนอง
3. รากเทียมไม่มีการขยับเมื่อเกิดการยึดติดกับกระดูกอย่างสมบูรณ์แล้ว
4. ในภาพรังสีรอบปลายรากเทียม ไม่พบเงาดำโปร่งรังสีรอบๆรากเทียมที่ฝัง

- ประเมินผลความสวยงามของฟันรากเทียมภายหลังการใช้งาน 1ปีโดยใช้ PES,WES score

- วัดผล periodontal status ได้แก่ probing depth, clinical attachment level, และ recession เปรียบเทียบกับฟันธรรมชาติคู่เทียบ

- วัด insertion torqueโดยใช้ ratchet with torque control device ภายหลังการฝังรากเทียม

- วัดความแม่นยำของตำแหน่งรากเทียมเมื่อฝัง one-piece ceramic โดยการใช้ Computer guided surgical template โดยประเมินจาก ตำแหน่งจริงของรากเทียมที่ถูกฝังโดยการใช้ computer guided surgical template เทียบกับตำแหน่งรากเทียมที่ได้รับการวางแผนมาก่อนหน้าผ่านโปรแกรม coDiagnostix โดยการใช้ภาพรังสีสามมิติทางทันตกรรม การประเมินความแม่นยำจะทำโดยการวัดความคลาดเคลื่อนของรากเทียมทั้งหมด 4 ตำแหน่ง ได้แก่ ความคลาดเคลื่อนของรากเทียมบริเวณส่วนต้นที่สุดของรากเทียมที่ฝังในกระดูก(mm.) ความคลาดเคลื่อนที่ตำแหน่งปลายสุดของรากเทียม (mm.) ความคลาดเคลื่อนในแนวความสูงของรากเทียม(mm.) และความคลาดเคลื่อนขององศารากเทียม

รายละเอียดการวัดความคลาดเคลื่อนนั้นจะกระทำโดยการนำ ตำแหน่งรากเทียมแท้จริงในกระดูกที่ได้จากภาพรังสีส่วนตัดอาศัยคอมพิวเตอร์ชนิดโคนบีมภายหลังจากการฝังรากเทียม มาซ้อนทับกับ ตำแหน่งของรากเทียมที่ได้วางแผนผ่านโปรแกรม coDiagnostix โดยมีตำแหน่งของฟันธรรมชาติซี่อื่นๆเป็นจุดอ้างอิง บันทึกความคลาดเคลื่อนบริเวณส่วนต้นสุดของรากเทียม บริเวณจุดปลายสุดของรากเทียม ความคลาดเคลื่อนแนวความสูง และความคลาดเคลื่อนขององศาของการฝังรากเทียม

**
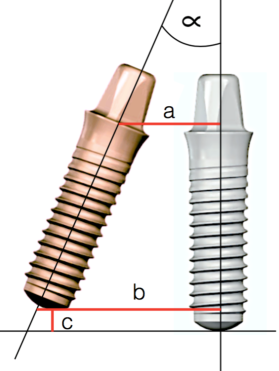
**

ระยะ a ;ความคลาดเคลื่อนของรากเทียมในส่วนต้น

ระยะ b ;ความคลาดเคลื่อนของตำแหน่งรากเทียมในส่วนปลาย

ระยะ c ;ความคลาดเคลื่อนของตำแหน่งรากเทียมแนวสูงต่ำ

มุม α ;ความคลาดเคลื่อนขององศาของรากเทียม

- การประเมินความปลอดภัย (Assessment of safety)

1. การถ่ายภาพรังสีส่วนตัดอาศัยคอมพิวเตอร์ชนิดโคนบีม

โดยเครื่อง 3D Accuitomo 170 (J.Morita, Kyoto, Japan)โดยใช้ 90kVp, 87.5mAs (5mA, 17.5s) โดยขนาด field of view (Height x Width) ที่ใช้สำหรับการวางแผนการฝังรากเทียมและภายหลังการฝังรากเทียมนั้นมีขนาดเท่ากับ 6x6 cm. โดยปริมาณรังสีที่ผู้เข้าร่วมวิจัยจะได้รับนั้นมีค่าประมาณ 62 µSvในตำแหน่งฟันหน้าในขากรรไกรบน^[3]^ และประมาณ 120 µSv ในตำแหน่งฟันหน้าในขากรรไกรล่าง^[3]^ การถ่ายภาพรังสีส่วนตัดอาศัยคอมพิวเตอร์ชนิดโคนบีมจะกระทำสองครั้งก่อนและภายหลังการรักษา นอกจากนี้การถ่ายภาพรังสีอื่นๆจะกระทำตามหลักการถ่ายภาพรังสีเพื่อการรักษาทางทันตกรรมรากเทียมทั่วไป ได้แก่ ภาพรังสีปริทัศน์ โดยในการถ่ายภาพรังสีปริทัศน์ผู้เข้าร่วมวิจัยจะได้รับปริมาณรังสีเท่ากับ 14.9 µSv^[4]^ และ ถ่ายภาพรังสีรอบปลายรากจะได้รับปริมาณรังสีเอกซ์เท่ากับ 9.5 µSv ^[4]^ต่อการถ่ายหนึ่งครั้ง ซึ่งในงานวิจัยนี้อาสาสมัครจะต้องถ่ายภาพรังสีรองปลายรากทั้งหมด 4 ครั้ง เป็นปริมาณรังสีที่ได้รับรากการถ่ายภาพรังสีรอบปลายรากเท่ากับ 38 µSv โดยเมื่อคำนวนแล้วพบว่า ปริมานรังสีที่คนไข้ได้รับนั้นมีค่าอยู่ในระดับที่ไม่เกินปริมาณรังสีที่กำหนดโดย สํานักงานปรมาณูเพื่อสันติ กระทรวงวิทยาศาสตร์และเทคโนโลยี ระบุว่าในบุคคลทั่วไปปริมาณรังสีเอกซเรย์ที่ได้รับไม่ควรเกิน
1 mSv ต่อปีโดยไม่รวมรังสีเอกซ์ที่ได้รับในชีวิตประจำวัน และผู้ป่วยจะได้รับการสวมเสื้อตะกั่วและปลอกคอกันรังสี

1. การวางแผนตำแหน่งการฝังรากเทียมผ่านโปรแกรม coDiagnostiX จะกระทำโดยทันตแพทย์ที่มีประสบการณ์การใช้โปรแกรมนี้และศึกษาเฉพาะทางด้านทันตกรรมรากเทียมโดยทุกๆการวางแผนนั้นมีการตรวจสอบตำแหน่งและความถูกต้องของรากเทียมอีกครั้งโดย อาจารย์ทันตแพทย์ผู้เชี่ยวชาญในการใช้โปรแกรม และช่างเทคนิคประจำบริษัท จากการทบทวนวรรณกรรม Kühl et al.2013^[19]^ แสดงให้เห็นว่าการใช้โปรแกรม coDiagnostiX ร่วมกับการขึ้นรูป surgical template โดยการใช้เครื่องพิมพ์สามมิติ 3D printing device มีความเที่ยงตรงและแม่นยำสูงในทุกๆมิติ โดยการศึกษาแสดงให้เห็นถึงค่าเฉลี่ยความคลาดเคลื่อนที่เกิดขึ้นของตำแหน่งรากเทียมเท่ากับ 0.22 mm. (โดยอยู่ในช่วง0.07-0.38 mm.) ซึ่งในทางคลินิคนั้นไม่แตกต่างกัน
2. การใช้ one-piece ceramic implant ในตำแหน่งฟัน central และ lateral incisor ในคนไข้ที่มีการสบฟันปกติจะพบว่ามี survival rate และ success rate 100%^[21]^ และ 98.3%^[20]^ ตามลำดับ จากข้อมูลนี้บ่งชี้ถึงความสำคัญในการคัดเลือกผู้ป่วยให้เหมาะสมจะก่อให้เกิดความล้มเหลวในระดับต่ำ โดยในงานวิจัยนี้การคัดเลือกอาสาสมัครนั้นจะกระทำอย่างรัดกุมตามเกณฑ์การคัดเข้าที่ได้กล่าวไป ในรายที่พบว่ามีสัญญาณบ่งถึงการสบฟันที่ไม่ปกติหรือมีการทำงานนอกหน้าที่ของระบบบดเคี้ยวจะถูกคัดออกจากงานวิจัยทันที โดยตลอดงานวิจัยนั้นจะมีการติดตามผลเป็นระยะอย่างสม่ำเสมอ ถ้าพบความผิดปกติของครอบฟันบนรากเทียม เช่นการแตกบิ่นของวัสดุครอบฟัน หรือ การละลายของกระดูกรอบรากเทียมที่บ่งชี้ถึงแรงสบฟันที่ผิดปกติอันเป็นสาเหตุให้เกิดการแตก ร้าว หรือการหักของ one-piece ceramic implant ในอนาคต การดำเนินการแก้ไขโดยการปรับการสบฟัน หรือการพิจารณาใส่เครื่องมือเพื่อช่วยป้องกันการทำงานนอกหน้าที่ของขากรรไกรจะกระทำโดยทันที นอกจากนี้ในกรณีที่เมื่อทำการเตรียมกระดูกเพื่อรองรับ one-piece ceramic implant แล้วพบว่า one-piece ceramic implant ที่ตำแหน่งนั้นจำเป็นต้องมีการกรอปรับแต่งรูปร่างของ abutment ให้มีความเหมาะสมต่อการบูรณะ อาสาสมัครจะถูกคัดออกจากงานวิจัยและทันตแพทย์จะทำการฝังรากเทียม Straumann bone level diameter 3.3 mm. แทนการใช้ one-piece ceramic implant

- สถิติหรือวิธีการอื่นๆที่ใช้ในการวิเคราะห์ข้อมูล (Data Analysis)

งานวิจัยนี้จะแสดงผล survival rate ภายใน 1 ปีในรูปแบบร้อยละ success rate ภายใน 1 ปี ใช้เกณฑ์อ้างอิงจาก Buser et at. 1990^[2]^ ซึ่งจะรายงานผลออกมาในรูปแบบร้อยละเช่นกัน ข้อมูลระดับ marginal bone ที่เปลี่ยนแปลงภายใน 1 ปีนั้นจะรายงานในรูปแบบค่าเฉลี่ยและส่วนเบี่ยงเบนปกติที่ระดับความเชื่อมั่น 95% นอกจากนี้ข้อมูลที่มีลักษณะต่อเนื่องได้แก่ probing depth , clinical attachment level และ recession จะบันทึกและเปรียบเทียบกับฟันคู่ธรรมชาติคู่เทียบในแต่ละช่วงเวลา นอกจากนี้ยังนำค่าที่ได้ในแต่ละช่วงเวลาของฟันรากเทียมมาเปรียบเทียบกัน โดยสถิติ Wilcoxon singed-rank test ประเมินด้วยโปรแกรม SSPS 17.0 for Windows (Chicago, IL, USA) ที่ระดับความเชื่อมั่นที่ 95%

งานวิจัยนี้มีการแสดงผลความแม่นยำของตำแหน่งรากเทียมโดยการวัดตำแหน่งจริงของรากเทียมภายหลังการฝังโดยการใช้ภาพรังสีส่วนตัดอาศัยคอมพิวเตอร์ชนิดโคนบีมเปรียบเทียบตำแหน่งของรากเทียมที่วางแผนไว้ในโปรแกรม coDiagnostiX (โปรแกรมสำหรับใช้วางแผนตำแหน่งของรากเทียม โดยการนำ ภาพรังสีส่วนตัดอาศัยคอมพิวเตอร์ชนิดโคนบีมก่อนการฝัง มา superimposition ด้วยกันกับแบบปูนจำลองสภาพช่องปาก ที่ได้รับการ scan ออกมาเป็นSTL. files จากนั้นเลือก one-piece ceramic implant ตามขนาดและความยาวที่ต้องการ ไปวางในตำแหน่งที่เหมาะสม) การเปรียบเทียบตำแหน่งจริงของรากเทียมและตำแหน่งที่ได้วางแผนใช้สถิติ t-test ที่ ระดับความเชื่อมั่นที่ 95%

14. กระบวนการเชิญชวนให้เข้าร่วมการวิจัย (Recruitment process) และกระบวนการขอความยินยอมให้เข้าร่วมการวิจัย (Informed consent process)

- 1. สถานที่ที่จะเข้าถึงอาสาสมัคร : ศูนย์รากเทียม คณะทันตแพทยศาสตร์ มหาวิทยาลัยมหิดล
  2. กระบวนการเข้าถึงและเชิญชวนอาสาสมัคร

ผู้ปฏิบัติงานทันตกรรมประจำศูนย์รากเทียม (นางสาว.นิชานันท์ แซ่ฮุ้น) จะเป็นผู้ประชาสัมพันธ์งานวิจัยให้แก่คนไข้ที่ถึงคิวทำรากเทียม เเละคนไข้ทั่วไปที่เดินมาติดต่อรับบริการที่ศูนย์ทันตกรรมรากเทียมโดยเจ้าหน้าที่จะได้รับการอธิบายให้ข้อมูลเกี่ยวกับงานวิจัยจาก ทพญ.นพรัตน์ สุขสด จนมีความเข้าใจและทราบถึงเกณฑ์ในการคัดเลือกอาสาสมัคร เมื่อมีผู้สนใจต้องการเข้าร่วมโครงการหรือต้องการทราบรายละเอียดเพิ่มเติม ทพญ. นพรัตน์ สุขสด จะเป็นผู้ให้ข้อมูลแก่ผู้สนใจโดยละเอียด การให้ข้อมูลนั้น ทพญ.นพรัตน์ สุขสด จะแจกแจงถึงที่มาและความสำคัญของงานวิจัย ข้อดีและความเสี่ยงที่อาจเกิดขึ้น รวมถึงระยะเวลาในการติดตามผลแก่คนไข้และให้คนไข้เป็นผู้ตัดสินใจและลงรายชื่อและเบอร์ติดต่อในการเข้าร่วมโครงการวิจัยได้ที่ผู้ปฏิบัติงานทันตกรรมประจำศูนย์รากเทียม (นางสาว.นิชานันท์ แซ่ฮุ้น)

สื่อช่วยประชาสัมพันธ์

☑ ไม่มี

- 1. กระบวนการขอความยินยอมให้เข้าร่วมการวิจัย (Informed consent process)

☑ ต่อเนื่องกับกระบวนการเชิญชวนให้เข้าร่วมการวิจัย (recruitment process)

- 1. เอกสารชี้แจงอาสาสมัคร (Volunteer information sheet) และหนังสือแสดงเจตนายินยอมเข้าร่วมการวิจัยโดยได้รับการบอกกล่าวและเต็มใจ (Informed consent form)

☑ มี

☑ เอกสารชี้แจงอาสาสมัครและหนังสือแสดงเจตนายินยอมเข้าร่วมการวิจัย แยกกันอย่างละ 1 ฉบับ

☑ สำหรับอาสาสมัครที่มิใช่ผู้เยาว์และสามารถตัดสินใจได้ด้วยตัวเอง

1. ข้อพิจารณาด้านจริยธรรมการวิจัยในคน (Ethical consideration)
   1. เหตุผลและความจำเป็นที่ต้องดำเนินการวิจัยในคน เนื่องจากมีงานวิจัยมากมายแสดงถึงประสิทธิภาพของone-piece ceramics implant ชนิด Yttria stabilised tetragonal zirconia polycrystalline (Y-TZP) ในห้องทดลองพบว่า Y-TZP implant นั้นมีค่า flexural strength,fracture toughness และค่า Young’s modulus ที่เหมาะสมต่อแรงบดเคี้ยวและการใช้งานในช่องปาก^[22]^ นอกจากนี้การทดลองในสัตว์ยังแสดงให้เห็นถึงการเกิด osseointegration ที่มีประสิทธิภาพที่ดีและเทียบเท่ากับ Titanium implant^[23]^ และในปัจจุบันงานวิจัยทางคลินิกในคนเกี่ยวกับความสำเร็จของone-piece ceramic implant เริ่มมีจำนวนมากขึ้นและผลที่ได้ก็แสดงให้เห็นถึงประสิทธิภาพของ one-piece ceramics implant แต่ในแง่ของการประเมิน PES และ WES ในบริเวณตำแหน่งที่มีผลต่อความสวยงามได้แก่ ในตำแหน่งฟัน central incisor และ lateral incisor นั้นยังไม่พบว่ามีผู้ทำการศึกษา ประกอบกับความเชื่อมั่นในการใช้ one-piece ceramics implant ในประเทศไทยยังอยู่ในระดับต่ำ ดังนั้นงานวิจัยนี้จึงมีประโยชน์ในแง่ของการประเมินประสิทธิภาพและความสวยงามที่เกิดขึ้น เพื่อเป็นการเพิ่มทางเลือกให้แก่คนไข้ และแสดงให้เห็นถึงการพัฒนาของศาสตร์ทันตกรรมรากเทียมที่เกิดขึ้นตลอดเวลา
   2. ประโยชน์ที่คาดว่าจะได้รับจากการวิจัยนี้ : ผู้เข้าร่วมวิจัยจะได้รับประโยชน์โดยตรงจากการเข้าร่วมการทำวิจัยโดยการวางแผนการผ่าตัดฝังรากเทียมผ่านโปรแกรม coDiagnostiX และขึ้นรูป guided surgical template ทำให้ตำแหน่งที่ผ่าตัดฝังรากเทียมนั้นมีความคลาดเคลื่อนของตำแหน่งรากเทียมน้อยกว่าเมื่อเทียบกับการฝังแบบ free hand จากการศึกษาของ Brief et al. 2005^[24]^ พบว่าความคลาดเคลื่อนของตำแหน่งรากเทียมที่ฝังด้วยมือเปล่านั้นจะอยู่ระหว่าง 1-1.5 mm. ซึ่งมีค่ามากกว่าเมื่อเทียบกับการใช้ computer guided surgical template นอกจากนี้ผลที่ได้จากการศึกษานี้จะแสดงให้เห็นถึงผลทางคลินิก และความสวยงามที่เกิดจากการ ceramics implant ในตำแหน่งที่มีผลต่อความสวยงาม ซึ่งจะเป็นการทดสอบประสิทธิภาพของ one-piece ceramic implant ทำให้ทันตแพทย์มีความมั่นใจในการเลือกใช้ ceramics implant มากขึ้น
   3. ความเสี่ยงที่อาจจะเกิดเหตุการณ์ไม่พึงประสงค์ต่ออาสาสมัคร
      1. เคยมีการวิจัยทำนองเดียวกับโครงร่างที่เสนอนี้มาก่อนหรือไม่ และเคยเกิดเหตุการณ์ไม่พึงประสงค์อย่างไร มีการศึกษามาตรการป้องกันและแก้ไขที่นักวิจัยเตรียมไว้ในโครงการนี้
         จากการทบทวนวรรณกรรมพบการศึกษาของ Jung et al 2015^[20]^ ที่กระทำการศึกษาทางคลินิกถึงผลของ one-piece ceramic implant ร่วมกับการบูรณะครอบชั่วคราวทันที (immediate provisionalization) ใน 1 ปี พบว่า survival rate เท่ากับ 98.3% นอกจากนี้ Grassi et al 2015^[25]^ ได้ทำการศึกษาผลทางคลินิกและการตอบสนองของเนื้อเยื่อรอบรากเทียบในระยะเวลา 5 ปี ได้ผลลัพธ์เป็นที่น่าพอใจเปรียบเทียบได้กับ titanium implant โดยเหตุการณ์ไม่พึงประสงค์ที่เกิดขึ้นคือ การหลุดของรากเทียมภายหลังการฝัง ซึ่งเกิดขึ้นในรายที่ทำการฝัง one-piece ceramic implant ภายหลังการถอนฟันทันทีร่วมกับการทำ immediate provisional และการศึกษายังพบอีกว่าโอกาสที่ one-piece ceramic implant จะหลุดนั้นเกี่ยวข้องกับประสบการณ์ของทันตแพทย์ผู้ทำการผ่าตัด การศึกษาของ Borgonovo et al. 2015^[21]^ พบว่ามี protocol ที่คล้ายคลึงกับงานวิจัยนี้ได้ผลลัพธ์ survival rate 100% ที่ระยะเวลาการติดตามผล 4 ปี

มาตรการการป้องกันเหตุการณ์ไม่พึงประสงค์ได้แก่ คัดเลือกคนไข้ให้ตรงตามเกณฑ์คัดเข้า ทันตแพทย์ผู้ทำการผ่าตัดเป็นผู้มีประสบการณ์และศึกษาระบบและขั้นตอนการฝัง one-piece ceramic implant มาแล้วเป็นอย่างดี การทำ immediate provisionalization จะกระทำในคนไข้ที่ได้ insertion torque มากกว่าหรือเท่ากับ 35 Ncm. เท่านั้น

มาตรการการแก้ไขหากเกิดเหตุการณ์ไม่พึงประสงค์ หัวหน้าโครงการวิจัยจะเป็นผู้แก้ไขและผ่าตัดฝังรากเทียมตัวใหม่ให้คนไข้ในกรณีที่เกิดการหลุด แตกหัก ของ one-piece ceramic implant และทำการบูรณะครอบฟันบนรากเทียมชิ้นใหม่ให้กลับมาใช้งานได้ตามปกติ

- - 1. ผู้รับผิดชอบค่าใช้จ่ายในการแก้ไข หรือศึกษาเหตุการณ์ไม่พึงประสงค์จากการวิจัย
       ในกรณีที่มีเหตุการณ์ไม่พึงประสงค์หรือความเสียหายต่ออาสาสมัครในโครงการ หัวหน้าโครงการวิจัยและผู้วิจัยร่วมจะเป็นบุคคลที่แก้ไขและรับผิดชอบความเสียหายที่เกิดขึ้น อาสาสมัครจะไม่ต้องเสียค่าใช้จ่ายในการแก้ไขในกรณีที่เกิดการแตกหัก หรือหลุดของรากเทียม
    2. ชื่อผู้รับผิดชอบหรือแพทย์ และหมายเลขโทรศัพท์ที่สามารถติดต่อได้ตลอดเวลา หากเกิดเหตุการณ์ไม่พึงประสงค์จากการวิจัย
       ทพ.ชัชชัย คุณาวิศรุต โทร 089-073-7030
       ทพญ. นพรัตน์ สุขสด โทร 083-543-5669
    3. กรณีเป็นการวิจัยทางคลินิก วิจัยมีวิธีการแจ้งแพทย์เจ้าของไข้ หรือ แพทย์อื่นที่ต้องมาให้การรักษาอาสาสมัครทราบว่าบุคคลผู้นั้นอยู่ในระหว่างดำเนินการวิจัยได้ด้วยวิธีใด

ในงานวิจัยนี้จะมีการระบุในแฟ้มประวัติของผู้ป่วยว่าคนไข้เป็นอาสาสมัครในโครงการวิจัยเพื่อการชี้แจงให้ทันตแพทย์ท่านอื่นทราบ นอกจากนี้ในส่วนของทันตแพทย์เจ้าของคนไข้นั้นจะทราบว่าคนไข้ของตนเองเข้าร่วมเป็นอาสาสมัครในโครงการตั้งแต่ครั้งแรกที่เริ่มทำการรักษาผ่านการชี้แจงจาก ทญฺนพรัตน์ สุขสด นอกจากนี้ทันตแพทย์เจ้าของคนไข้จะได้รับทราบถึงขึ้นตอนที่เพิ่มมาจากการฝังรากเทียมปกติ จาก ทญ นพรัตน์

- 1. หลักฐานหรือข้อมูล (เอกสารอ้างอิง)

จากการทบทวนวรรณกรรมพบว่ามีศึกษาในห้องปฎิบัติการหลายฉบับพบว่า one-piece ceramic implant ชนิด Y-TZP นั้นมีคุณสมบัติทางกายภาพและคุณสมบัติทางชีวภาพที่เหมาะสมต่อการใช้เป็นวัสดุรากเทียม^[3, 6, 8]^ นอกจากนี้การศึกษาในสัตว์ทดลองยังแสดงให้เห็นถึงความปลอดภัยของวัสดุที่มีต่อเนื้อเยื่อ และการเกิดการยึดติดของกระดูกที่บริเวณพื้นผิวรากเทียมที่มีประสิทธิภาพดีเทียบเท่าหรือมากกว่าวัสดุไทเทเนียม^[9, 10, 26]^ นอกจากนี้การศึกษาทางคลินิกหลายฉบับแสดงให้เห็นถึงประสิทธิผลของ one-piece ceramic implant ในการใช้งานจริง ผลลัพธ์ที่เกิดขึ้นนั้นเป็นที่น่าพอใจทั้งในแง่ของการยึดติดในกระดูก การตอบสนองของเนื้อเยื่อรอบๆ one-piece ceramic implant และความสวยงาม^[20, 21, 27]^ นอกจากนี้ไม่พบว่ามีรายงานอุบัติการณ์การสูญเสียรากเทียมอันเนื่องมาจากการปฏิเสธของภูมิคุ้มกันของร่างกาย ดังนั้นการใช้ one-piece ceramic implant ในงานวิจัยนี้จึงมีความปลอดภัย

- 1. วิธีการปกป้องความลับข้อมูลส่วนตัวของอาสาสมัคร

☑ ใช้รหัสแทนชื่อและข้อมูลส่วนตัวของอาสาสมัครในการบันทึกข้อมูลในแบบเก็บข้อมูล

☑ มีการบันทึกข้อมูลเป็น ☑ รูปถ่าย โดยผู้ที่สามารถเข้าถึงข้อมูลรูปภาพได้แก่ทันตแพทย์ผู้ที่เป็นเจ้าของเคสนั้นๆ และรูปถ่ายจะไม่มีการเปิดเผยใบหน้าและได้รับอนุญาตจากอาสาสมัครก่อนเท่านั้นและเมื่อสิ้นสุดการวิจัย จะทำลายเอกสารที่สามารถสืบค้นไปยังผู้ป่วยได้ด้วย เครื่องทำลายเอกสารชนิดย่อยเป็นเส้น

1. เอกสารที่แนบมาพร้อมแบบเสนอโครงการวิจัย ได้แก่ (โปรดทำเครื่องหมาย☑) และรายการที่ไม่ได้แนบขอให้ตัดออก

☑ แบบเสนอโครงการวิจัย (Submission Form) ต้นฉบับ 1 ชุด สำเนา 3 ชุด รวมเป็น 4 ชุด พร้อมไฟล์อิเล็กทรอนิกส์

☑ โครงร่างวิจัย (Protocol/Proposal) ต้นฉบับ 1 ชุด สำเนา 3 ชุด รวมเป็น 4 ชุด พร้อมไฟล์ (กรณีมีทุนวิจัยหรือเป็นโครงการวิจัยของนักศึกษา)

☑ เอกสารชี้แจงอาสาสมัคร (Volunteer Information Sheet) จำนวน 20 ชุด พร้อมไฟล์อิเล็กทรอนิกส์

☑ หนังสือแสดงเจตนายินยอมเข้าร่วมการวิจัยโดยได้รับการบอกกล่าวและเต็มใจ (Informed Consent Document) จำนวน 20 ชุด พร้อมไฟล์อิเล็กทรอนิกส์

☑ ประวัติส่วนตัว ตำแหน่ง สถานที่ทำงาน และผลงานของหัวหน้าโครงการวิจัย (Principal Investigator’s Curriculum Vitae) จำนวน 4 ชุด

☑ หนังสือรับรองว่าจะเริ่มดำเนินการวิจัยภายหลังจากได้รับการรับรองจากคณะกรรมการจริยธรรมการวิจัยในคนประจำคณะทันตแพทยศาสตร์และคณะเภสัชศาสตร์ มหาวิทยาลัยมหิดล ต้นฉบับ 1 ชุด สำเนา 3 ชุด รวมเป็น 4 ชุด

☑ แบบบันทึกข้อมูลที่จะใช้ในการวิจัย (Case Report Form/Case Record Form) โปรดระบุ จำนวน 4 ชุด พร้อมไฟล์อิเล็กทรอนิกส์

❏ หลักฐานการจ่ายค่าธรรมเนียมหรือหลักฐานการขออนุเคราะห์ยกเว้นค่าธรรมเนียม

1. ข้อสัญญา
2. ข้าพเจ้าและคณะนักวิจัยดังมีรายนามและได้ลงชื่อไว้ในเอกสารนี้ จะดำเนินการวิจัยตามที่ระบุไว้ในโครงการวิจัยฉบับที่ได้รับการรับรองจากคณะกรรมการจริยธรรมการวิจัยในคนประจำคณะทันตแพทยศาสตร์และ คณะเภสัชศาสตร์ มหาวิทยาลัยมหิดล และได้ขอความยินยอมจากอาสาสมัครอย่างถูกต้องตามหลักจริยธรรมการวิจัยในคนดังที่ได้ระบุไว้ในแบบเสนอโครงการวิจัย โดยจะให้ความเคารพในสิทธิ และคำนึงถึงสวัสดิภาพของอาสาสมัครเป็นสำคัญ
3. หากมีความจำเป็นต้องปรับแก้ไขโครงการวิจัย ข้าพเจ้าจะแจ้งให้คณะกรรมการจริยธรรมฯ (MU-DT/PY-IRB) เพื่อขอรับการพิจารณารับรองก่อนเริ่มดำเนินการปรับเปลี่ยนทุกครั้ง และหากการปรับโครงการวิจัยมีผลกระทบต่ออาสาสมัคร ข้าพเจ้าจะแจ้งการปรับเปลี่ยนและขอความยินยอมจากอาสาสมัครทุกครั้ง
4. ข้าพเจ้าจะรายงานเหตุการณ์ไม่พึงประสงค์/เหตุการณ์ที่ไม่สามารถคาดเดาได้ล่วงหน้าในระหว่างการวิจัย ตามระเบียบของคณะกรรมการจริยธรรมฯ (MU-DT/PY-IRB) ภายในเวลาที่กำหนด และจะให้ความช่วยเหลือในการแก้ไขเหตุการณ์ไม่พึงประสงค์ที่เกิดขึ้นระหว่างการวิจัยอย่างเต็มความสามารถ
5. ข้าพเจ้าจะรายงานผลการดำเนินการวิจัยประจำปี หรือตามที่คณะกรรมการจริยธรรมฯ กำหนด
6. ข้าพเจ้าและคณะนักวิจัยมีความรู้ความเข้าใจในกระบวนการวิจัยที่เสนอมาอย่างดีทุกขั้นตอน และมีความสามารถในการแก้ไขปัญหา หรือเหตุการณ์ไม่พึงประสงค์ที่อาจจะเกิดขึ้นในระหว่างการวิจัย เพื่อความปลอดภัยและ สวัสดิภาพของอาสาสมัครได้เป็นอย่างดี

ลงชื่อ...............................................หัวหน้าโครงการวิจัย

(...............................................)

วันที่…………/……………/....…..

ลงชื่อ...............................................นักวิจัยร่วม

(...............................................)

วันที่…………/……………/....…..

ลงชื่อ...............................................นักวิจัยร่วม

(...............................................)

วันที่…………/………………/……..

1. การรับรองจากหัวหน้าหน่วยงานหรือผู้บังคับบัญชาโดยตรงหรืออาจารย์ผู้ควบคุมวิทยานิพนธ์ที่อนุมัติให้ดำเนินการวิจัยได้

ลงชื่อ......................................................

(.....................................................)

หัวหน้าหน่วยงาน

วันที่…………/……………/....…..

เอกสารอ้างอิง

1.Jung R.E., Grohmann P., Sailer I., Steinhart Y.N., Feher A., Hammerle C., Strub J.R.& Kohal R.(2015) Evaluation of a one-piece ceramic implant used for single-tooth replacement and three-unit fixed partial dentures: a prospective cohort clinical trial.*Clin Oral Implants Res*.

2.Buser D., Mericske-Stern R., Bernard J.P., Behneke A., Behneke N., Hirt H.P., Belser U.C.& Lang N.P.(1997) Long-term evaluation of non-submerged ITI implants. Part 1: 8-year life table analysis of a prospective multi-center study with 2359 implants.*Clin Oral Implants Res*;8(3):161-172.

3.Ludlow J.B., Timothy R., Walker C., Hunter R., Benavides E.& Samuelson D.B.(2015) Correction to Effective dose of dental CBCT--a meta analysis of published data and additional data for nine CBCT units.*Dentomaxillofac Radiol*;44(7):20159003.

4.Ludlow J.B.(2008) Regarding "Influence of CBCT exposure conditions on radiation dose".*Oral Surg Oral Med Oral Pathol Oral Radiol Endod*;106(5):627-628; author reply 628-629.
